# Dual-Grafting of Microcrystalline Cellulose by Tea Polyphenols and Cationic ε-Polylysine to Tailor a Structured Antimicrobial Soy-Based Emulsion for 3D Printing

**DOI:** 10.1021/acsami.1c19430

**Published:** 2022-04-27

**Authors:** Mahdiyar Shahbazi, Henry Jäger, Rammile Ettelaie

**Affiliations:** † Institute of Food Technology, University of Natural Resources and Life Sciences (BOKU), Muthgasse 18, Vienna 1190, Austria; ‡ Food Colloids and Bioprocessing Group, School of Food Science and Nutrition, University of Leeds, Leeds LS2 9JT, U.K.

**Keywords:** Pickering emulsion, surface hydrophobicity, bioactivity properties, interfacial adsorption behavior, pseudoplasticity, thixotropic feature, mechanical property, 3D printing, toughening mechanism

## Abstract

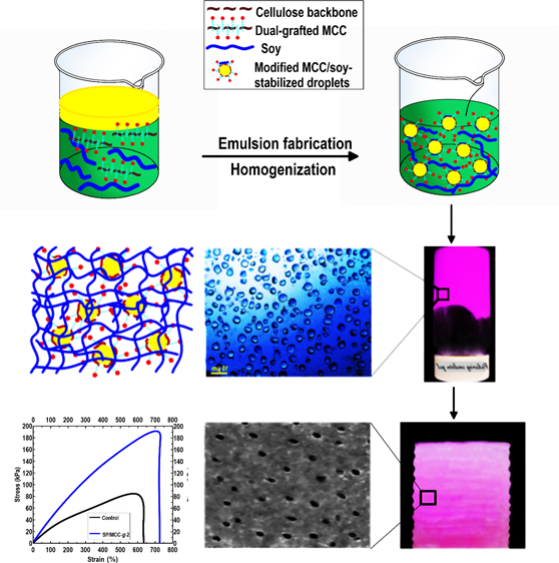

An imperative processing way to produce 3D printed structures with enhanced multifunctional properties is printing inks in the form of a gel-like colloidal emulsion. The surface-modified microcrystalline cellulose (MCC) is an excipient of outstanding merit as a particulate emulsifier to manufacture a stable Pickering emulsion gel. The tuning of the MCC structure by cationic antimicrobial compounds, such as ε-polylysine (ε-PL), can offer a surface activity with an antimicrobial effect. However, the MCC/ε-PL lacks the appropriate emulsifying ability due to the development of electrostatic complexes. To overcome this challenge, (i) a surface-active MCC conjugate was synthesized by a sustainable dual-grafting technique (ii) to produce a highly stable therapeutic soy-based Pickering emulsion gel (iii) for potential application in 3D printing. In this regard, the tea polyphenols were initially introduced into MCC by the free-radical grafting method to decrease the charge density of anionic MCC. Then, the antioxidative MCC-*g*-tea polyphenols were reacted by ε-PL to produce a dual-grafted therapeutic MCC conjugate (micro-biosurfactant), stabilizing the soy-based emulsion system. The results indicated that the dual-grafted micro-biosurfactant formed a viscoelastic and thixotropic soy-based emulsion gel with reduced droplet size and long-term stability. Besides, there was an improvement in the interfacial adsorption features of soy–protein particles after micro-biosurfactant incorporation, where the interfacial pressure and surface dilatational viscoelastic moduli were enhanced. Consequently, it was revealed that the therapeutic Pickering emulsion gel was more suitable to manufacture a well-defined 3D architecture with high resolution and retained permanent deformation after unloading (i.e., a recoverable matrix). This work established that the modification of the MCC backbone by tea polyphenols and ε-PL advances its bioactive properties and emulsifying performance, which finally obtains a soy-based 3D printed structure with noteworthy mechanical strength.

## 1 Introduction

In recent years, three-dimensional (3D) printing has been developed to fabricate custom-designed and robust 3D structures for a variety of bioengineering, pharmaceutical, and food applications.^1,2^ The extrusion-based dispensing module is the most extensively employed system in additive manufacturing, giving the rapid prototyping and processability of various biomaterials at a low cost.^2,3^ The successful utilization of extrusion-based 3D printing is typically associated with the progress of an effective printable ink with reinforced flow behavior.^4^ In this regard, gel-forming ability, dispersion stability, and rheological properties of the structured inks are the main functional features behind the extensive use of emulsion gels in 3D printing.^2^ In emulsion gels, the emulsified oil droplets are entrapped within 3D networks induced by cross-linking biopolymers.^5^ In various 3D printing applications, the stability of emulsion gels against physical deformation such as coalescence and gravitational separation is of great importance to retain their desired functional properties.^6^

Soy proteins are commonly used in 3D printing applications because of the desired gel-forming ability, which can develop an elastic structure.^2,7^ However, thanks to the high molecular weight, low solubility, and dense globular structure, they show a poor emulsification property compared to other derived proteins.^8,9^ The application of particulate (i.e., Pickering)-type emulsifiers is an imperative processing way to stabilize soy-based emulsions.^10,11^ Depended on a variety of solid particles, the development of Pickering emulsion gels shows considerable benefits in promoting pseudoplasticity, viscoelasticity, and long-term stability against flocculation/coalescence.^10,12^

Microcrystalline cellulose (MCC), as a rod-shaped fine crystal, is developed by acidic treatments of native cellulosic compounds under controlled hydrolysis circumstances.^12^ It is extensively applied in pharmaceutical and food applications because of biodegradability, low cost, relatively large specific surface area, and exclusive physicochemical properties.^12,13^ Previously, MCC was considered a hydrophilic polysaccharide with high charge density, lacking the effective emulsifying ability owing to the formation of strong agglomerates or poor miscibility.^12,13^ Therefore, the surface modification of MCC by the addition of specific molecules and polymers decreases the particles’ aggregation and improves colloidal stability, which reinforces its emulsifying ability.^10,12,13^ As MCC particles are only stable in aqueous solutions with low ionic strength, the chemical modification of their surface properties by covalent chemistry is commonly an “un-green”, lengthy, and costly approach. It was reported that free-radical grafting is an efficient sustainable reaction to introduce active compounds onto the microparticles’ backbone. This technique typically employs the ascorbic acid/hydrogen peroxide redox pair as an initiator,^13^ which is much less toxic than other chemical treatments. This method also avoids the oxidation of bioactive compounds, which becomes a perfect technique to synthesize the grafted microparticles aimed at use in the healthcare and food sector.^10^

There is an increasing awareness that the constant extensive usage of chemical compounds to inhibit bacterial growth in consumer products and industry poses a serious health threat.^14^ Thus, it is important to consider natural compounds to treat infectious diseases. As a natural cationic antimicrobial compound, ε-polylysine (ε-PL) is a homopolymer including l-lysine monomers linked with isopeptide linkages between α-carboxyl and ε-amino groups. It is extremely efficient against a broad range of spoilage organisms and pathogens. The cationic ε-PL also has an emulsification property because of the existence of the amino groups along its backbone, which induces a positive charge in water. In compositionally complex systems, its antimicrobial properties and emulsifying ability can be highly affected by electrostatic interactions with other anionic molecules, such as MCC.^15^ As a result, some insoluble compounds with anionic species are formed in the system, resulting in the development of coacervates. This inhibits the surface activity of ε-PL and also decreases its minimum inhibitory concentration against pathogens.^16^ A possible strategy to reduce the coacervation development can be decreasing the charge density of anionic molecules and/or interaction of primary amine groups (−NH_2_) of ε-PL with quinones through a Schiff-base and/or Michael addition reaction. This can reduce the electrostatic complexes, which achieves a balance between high emulsion stability and therapeutic effectiveness.

Well-known for their antioxidant and antibacterial properties, tea polyphenols (TPs) show a variety of applications in the pharmaceutical and food fields.^17^ Catechin compounds are the predominant compounds in green tea, which are considered flavan-3-ols. Other common types of catechins comprise (−)epicatechin (EC), (−)epicatechin-3-gallate (ECG), (−)epigallocatechin (EGC), and (−)epigallocatechin-3-gallate (EGCG). Despite the remarkable therapeutic potentials, TPs suffer from several limitations such as instability once subjected to heat, light, and basic environments, providing poor bioavailability with a fast metabolism. These disadvantages hinder the clinical application of TPs.^18^ Reportedly, free-radical grafting can endow MCC with the antioxidant properties of polyphenols, enhancing the bioavailability of polyphenols, and consequently improving the therapeutic effects of MCC.^10^ Moreover, the hydrophobic MCC-*g*-polyphenol compound, with a reduced charge density, develops quinones, which can react with −NH_2_ of ε-PL through a Schiff-base and/or Michael addition reactions. This effectively reduces the electrostatic complexes and enhances the surface activity of MCC, while also increasing its therapeutic effect.^13,19^ In our recent work,^20^ we used gallic acid to decrease the charge density of the anionic MCC, which reduced the electrostatic complexes between the anionic MCC and a cationic antimicrobial compound, that is, lauric arginate.

In this study, we aimed to enhance the surface activity and medicinal effects of MCC by using cationic ε-PL. To decrease the development of complex coacervates between MCC and ε-PL, first, TPs were grafted onto the MCC backbone through the free-radical grafting method to reduce the formation of large aggregates and electrostatic complexes, as well as enhance the antioxidant properties of MCC. Next, ε-PL was interacted by the TPs coated-MCC conjugate through the Schiff base reaction and/or Michael addition to produce a multifunctional dual-grafted MCC. Finally, the multifunctional grafted MCCs (micro-biosurfactant) were used to stabilize a soy-based emulsion, where the produced Pickering emulsion gel was printed via an extrusion-based 3D printing system to develop a therapeutic protein-based 3D printed object.

## 2 Experimental Section

### 2.1 Dual-Grafting Modification of MCC

Initially, a free-radical grafting method was applied to graft the TPs onto the MCC backbone (see Supporting Information, Section S.2.1).^10^ In this case, the hydrogen peroxide comprising ascorbic acid was introduced into the MCC-based dispersion through an ultrasonic cleaning device (Bandelin 400, Berlin, Germany).^10^ The product was labeled as MCC-*g*-TP. In the final step, the ε-PL was added to the TP-coated MCC conjugate in an ambient temperature and atmosphere.^20^ Afterward, this product (MCC-*g*-TP-*g*-PL) was centrifuged (Eppendorf centrifuge 5417R, Hamburg, Germany) and freeze-dried to form a well-separated particle. Likewise, an MCC/TP was developed without a redox initiator compound. For a better interpretation of the reference and detailed preparation of control MCC, MCC/TP, MCC-*g*-TP, and MCC-*g*-TP-*g*-PL in this section, the reader is referred to the Supporting Information of this article (Section S.2).

### 2.2 Preparation of Soy Protein-Based Pickering Emulsion

An O/W emulsion was developed by blending 10 wt % sunflower oil and 90 wt % aqueous soy protein isolate (SPI)-based dispersions using a two-stage high-pressure Microfluidizer processor (M110-PS, Microfluidics international Corp., Newton, MA) (see Supporting Information, Section S.3).^10,20^ The full-fat stabilized emulsion, considered as control henceforth, was utilized to manufacture the reduced-fat emulsions. Different reduced-fat SPI-based Pickering emulsion gels were prepared by replacing oil with the stock suspensions of pristine MCC (SP/MCC), MCC-*g*-TP (SP/MCC-*g*-1), and MCC-*g*-TP-*g*-PL (SP/MCC-*g*-2). The detailed information regarding the development of Pickering emulsion gels could also be found in the Supporting Information of this article (Section S.3).

### 2.3 3D Printing of Prepared Pickering Emulsion Gels

The prepared soy protein-based inks were printed through an extrusion-based 3D printer (nScrypt-3D-450, nScrypt, Orlando, FL), connected to a syringe pump (PHD Ultra; Harvard Apparatus Holliston, MA). A special cube shape was modeled by the application of computer-aided design software (AutoCAD; Autodesk Inc., San Rafael, CA), and converted into an STL file.^4^ Each Pickering emulsion gel was then printed in a size of (5 × 5 × 5) cm^3^ as a cube through a needle diameter of 1 mm with an extrusion flow speed of 50 mL min^–1^ at an ambient temperature on a special plastic surface.^20^ Detailed information on the printing procedures has been provided in the Supporting Information of this article (Sections S.4.9 and S.8).

### 2.4 Characterizations and Calculation Details

All the information concerning the characterization techniques and various factors to be analyzed were very similar to those included in our previous publications.^10,20^ The detailed materials, synthetic process, and the characterizations of modified MCC, Pickering emulsion gels, and 3D printed structures are described in the Supporting Information.

## 3 Results and Discussion

### 3.1 Characterization of Grafted MCCs (the Micro-Biosurfactants)

**Scheme 1:**
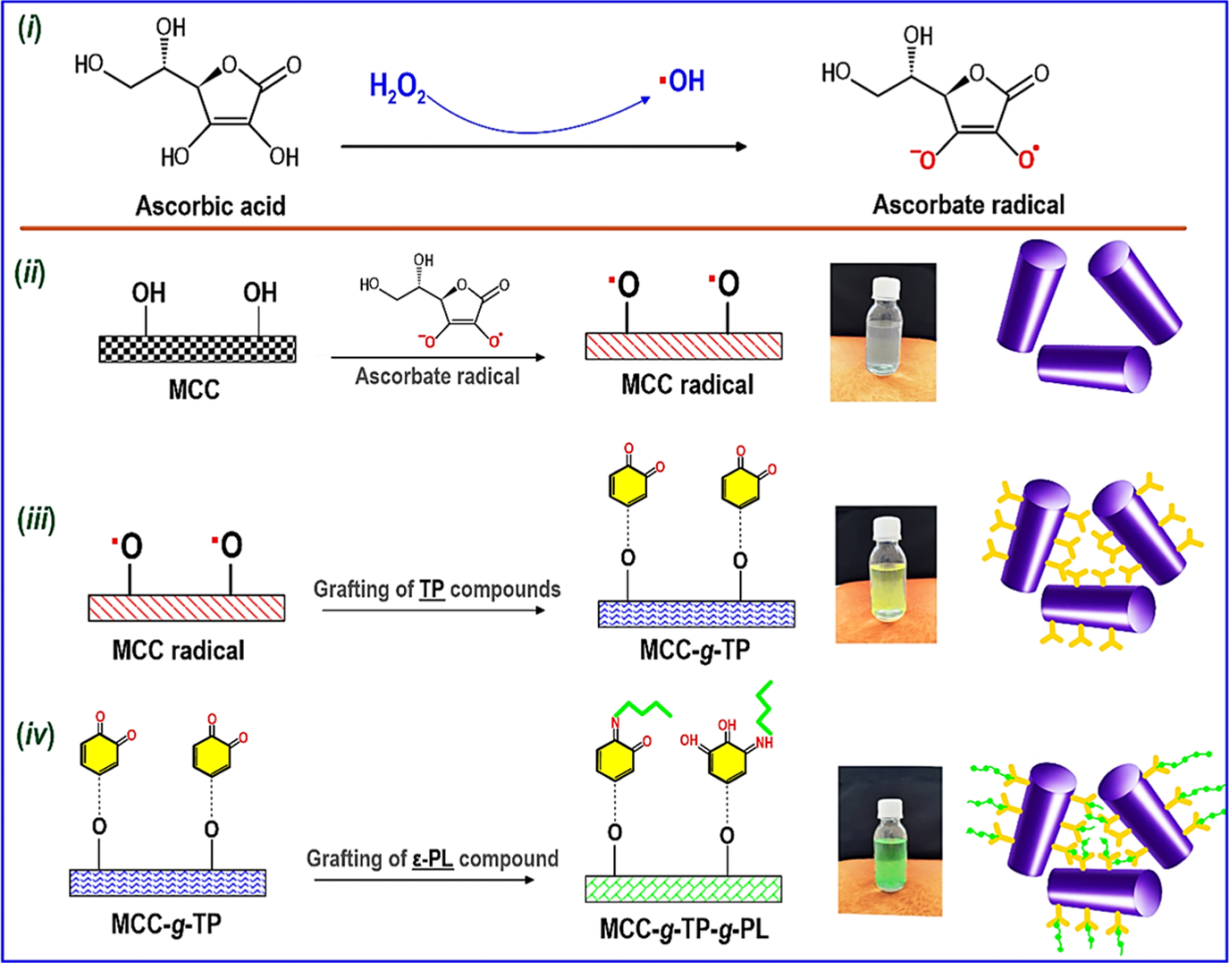
Schiff-Base Reaction and/or Michael Addition Products of Grafting Reactions of MCC by TPs and ε-PL The dotted line signifies the TPs grafting onto MCC via covalent and/or other types of intermolecular associations.

The pristine MCC powder used in this study was commercially labeled as Avicel PH-101, which is stable in an aqueous suspension as its surface sulfate half ester groups impart electrostatic repulsion.^10,13^ The pristine freeze-dried MCC powders were acquired in the neutral sodium form (Supporting Information, Figure S1), where it was simply re-dispersed in the aqueous solution using ultrasound (Supporting Information, Figure S1). The grafting of TPs onto MCC through the free-grafting process using the hydrogen peroxide/ascorbic acid redox pair (Scheme 1, rows i and ii) resulted in coated MCC (MCC-*g*-TP), preserving the colloidal stability of MCC with a somewhat yellow discoloration (Scheme 1, row iii). The grafting of ε-PL onto the TP-coated MCC (MCC-*g*-TP-*g*-PL) in the second phase caused the quick particle agglomeration and phase separation with a green discoloration (Scheme 1, row iv), suggesting an increased hydrophobicity after the dual-grafting process.^20^ The green agglomerated phases of MCC-*g*-TP-*g*-PL were collected and oven- or freeze-dried to develop a green powder. The obtained powder was homogeneously re-dispersed in toluene using an ultrasound-assisted method^20^ with no agglomeration (Supporting Information, Figure S1).

The reaction mechanism for the dual-grafting method is anticipated to pursue those described previously regarding other types of polyphenols and ε-PL on different substrates.^10,13,20^ It is assumed that the interaction between MCC and oxidized polyphenols led to the development of newly formed covalent linkages, as well as the formation of additional intermolecular interactions including π–π interactions, metal chelation, or hydrogen bonds.^10^ In the first stage, TPs interact with MCC at pH 8.5 as in this circumstance, the polyphenol oxidation and oligomerization are recognized to happen, especially with the adequate accessible dissolved oxygen.^20^ This results in the development of a high-molecular-weight quinone species with a reduced solubility. Apparently, the reduced solubility of TPs along with their intrinsic affinity to the cellulosic substrate causes the surface grafting of the polyphenols onto the MCC backbone.^10,13,20^ In the subsequent phase of the treatment, there is a reaction of the quinone with the primary amine groups of ε-PL through the Schiff-base and/or Michael addition reactions (Scheme 1).

#### 3.1.1 FTIR Measurement

**Figure 1:**
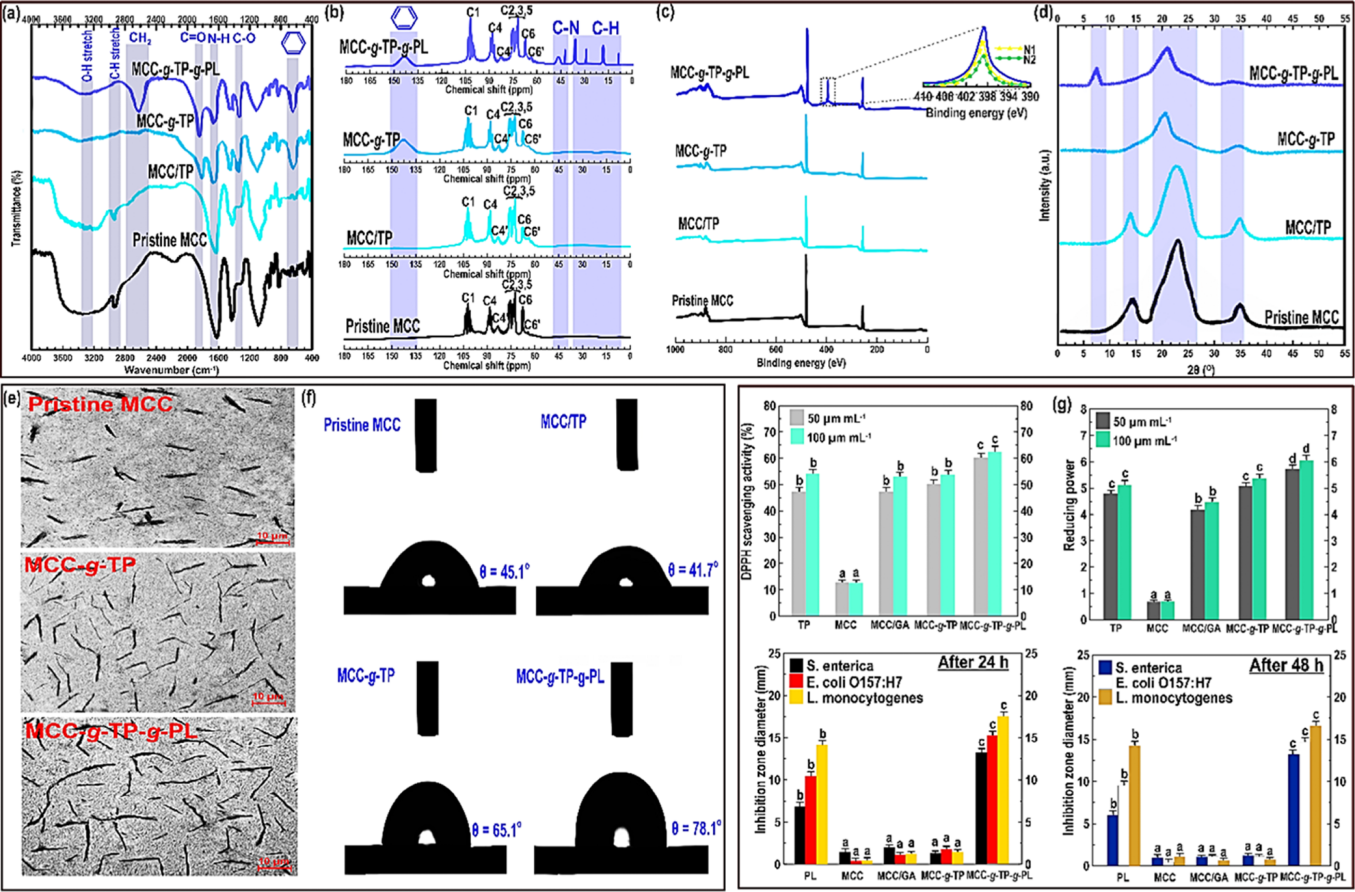
Characterizations of different products of grafted MCC conjugates. (a) FTIR, (b) ^13^C NMR, (c) XPS, (d) XRD, (e) TEM, (f) water contact angle, and (g) bioactive properties. In the case of bioactive features, the means inside each column with various letters (a–d) are significantly different (*P* < 0.05) according to Duncan’s test.

Compared to the FTIR spectrum of pristine MCC (Supporting Information, Section S.6.1), the hydroxyl (-OH) stretching (∼3350 cm^–1^) was reduced in MCC-*g*-TP (Figure 1a), representing that a conjugation reaction occurred at the −OH sites of the MCC backbone. Besides, Figure 1a shows that the C–H stretch vibration of −CH_3_ (∼2950 cm^–1^) was disappeared. This signifies that the hydrogen of −CH_3_ or −OH on the MCC backbone effectively interacted with the oxygen of −OH groups located at the TP through a hydrogen interaction. Moreover, there is the formation of a new strong carbonyl stretching vibration (C=O) at about 1860 cm^–1^, which simply confirmed the development of a hydrophobic surface. Another piece of evidence to prove the TP grafting onto the MCC was the emergence of the C–O stretching vibration band at about 1350 cm^–1^.^6,10,20^ Besides, there is an appearance of an obvious peak around 690 cm^–1^ resulting from the distortion vibrations of benzene rings.^10,20^ These observations suggested the interactions between MCC and TP through free-radical reaction.^10^

With regard to the FTIR spectrum MCC-*g*-TP, the peak intensity of the −OH stretching in the MCC-*g*-TP-*g*-PL was wider and more subdued (Figure 1a). This could be due to consuming more hydroxyl groups in the MCC backbone as affected by the Schiff base reaction and/or Michael addition.^20^ At the same time, the appearance of an asymmetrical and symmetrical −CH_2_ stretches from an alkyl chain around 2650 cm^–1^ suggested the grafting of ε-PL onto the MCC-*g*-TP. The dual-grafted micro-biosurfactant (i.e., MCC-*g*-TP-*g*-PL) also showed the presence of the C–O stretching band around 1350 cm^–1^ and a typical secondary N–H bending at about 1625 cm^–1^, proofing the incidence of a Michael addition.^20^

#### 3.1.2 ^13^C NMR Spectroscopy

Compared to the NMR spectrum of pristine MCC (Supporting Information, Section S.6.2), a new wide peak between 135 and 150 ppm was developed regarding MCC-*g*-TP and MCC-*g*-TP-*g*-PL (Figure 1b). This is caused by the aromatic rings of the phenolic acids,^10,13^ which agreed well with the characteristic FTIR band at 690 cm^–1^. The changes in the NMR spectrum of MCC-*g*-TP-*g*-PL were more marked in comparison with the MCC-*g*-TP. In this case, there is the formation of new strong resonances on the NMR spectrum of MCC-*g*-TP-*g*-PL, offering the presence of several pronounced peaks around 8.5, 13.5, 25.0, 36.2, 42.8, and 47.1 ppm (Figure 1b). These changes could be caused by the hydrocarbons, that is, the secondary carbon (−CH_2_−) and the primary carbon groups (−CH_3_).^20^ The obtained results further verified the dual-grafting of MCC-*g*-TP with ε-PL. On the other hand, the crystallinity obtained by ^13^C NMR offered that the crystallinity index of the pristine MCC (∼84%) slightly increased after the grafting treatments (∼87%).

#### 3.1.3 XPS Experiment

Figure 1c compares the XPS spectra of neat MCC, MCC/TP, MCC-*g*-TP, and MCC-*g*-TP-*g*-PL to further support the grafting reactions. Obviously, the XPS pattern of the neat MCC mainly includes oxygen and carbon.^20^ Concerning MCC/TP and MCC-*g*-TP samples, their patterns were comparable to the pristine MCC, in which no noticeable new peaks appeared on the XPS spectra. In contrast, ε-PL grafting onto MCC-*g*-TP induced a nitrogen band on the XPS spectrum of MCC-*g*-TP-*g*-PL. The resolving of this band (399.2 eV) produced two components, including an N1 peak at about 398.2 eV (related to aromatic C=N) and an N2 peak around 400.2 eV (assigned to aromatic C–N).^21^ These peaks clearly denote that both Schiff-base reaction and Michael addition in the MCC-*g*-TP-*g*-PL were developed.^20,22^ Additionally, the intensity of the characteristic peaks around 264.2 and 471.8 eV was notably increased, which were associated with the aromatic C=N and aromatic C–N, respectively.^20^ This further suggests that the ε-PL was efficiently grafted onto the MCC-*g*-TP backbone. According to the atomic proportions, the theoretical ratio of O to C regarding pure cellulose was reported about 0.83.^20^ In this work, the calculated ratios of O/C obtained by the XPS experiment were measured to be 0.79, 0.78, 0.77, and 0.49 concerning the pure MCC, MCC/TP, MCC-*g*-TP, and MCC-*g*-TP-*g*-PL, respectively. A huge decrease in the O/C ratio of the dual-grafted MCC conjugate was due to the presence of R-NH_2_ groups on the ε-PL backbone with no oxygen. It should be emphasized that a calculated difference in the ratio of O/C concerning pristine MCC and MCC-*g*-TP is likely due to some degree of contamination.^20^

#### 3.1.4 XRD Pattern

The diffractogram of MCC-*g*-TP obviously shows that the grafting of TP onto the MCC through the free-radical grafting reaction led to the disappearance of the typical XRD peak of MCC at about 2θ = 14.2° (Figure 1d). This signifies the successful interaction of TP with MCC in the inter-helical structure.^10^ The calculated relative crystallinity obtained by the XRD experiment showed that the residual-current device (RCD) of MCC decreased from an initial value of 78 to 52% after the development of MCC-*g*-TP. This denotes that the intensity of the characteristic peaks of MCC in the semi-crystalline regions was noticeably declined. Furthermore, there is a shift in the characteristic reflection of MCC from 2θ = 23.0° to 2θ = 20.5°. This specifies that the gallery spacing from *d*_001_ = 4.9 Å (2θ = 23.0°) increased to *d*_001_ = 5.2 Å (2θ = 20.5°) (Supporting Information, Section S.6.3). In this case, the functional groups of MCC most likely interacted with TP, leading to a change in the spatial structure of MCC. After the synthesis of MCC-*g*-TP-*g*-PL, the magnitude of MCC characteristic reflections was reduced more, coinciding with an important decrease of RCD to a level of 42%. Interestingly, a new typical reflation around 2θ = 7° (*d*_001_ = 6.3 Å) appeared on the diffraction pattern of MCC-*g*-TP-*g*-PL. This indicates the development of a newly formed crystalline domain in the amorphous area of MCC on account of the dual-grafting reaction.^20^

#### 3.1.5 Morphological Assessment

A transmission electron microscopy (TEM) investigation was used to monitor the morphological properties of different synthesized microparticles (Figure 1e). The particles in the pristine MCC and MCC/TP were in the micron range with a size of about 1–25 μm (Figures 1e and S3 in the Supporting Information). After the free-radical grafting and dual-grafting processes, there is an agglomeration of the particles, in which MCC-*g*-TP and MCC-*g*-TP-*g*-PL formed a larger particle with greater dimensions. As illustrated in the TEM image, the surface of the pristine MCC seemed somewhat smooth, while the grafted MCC conjugates showed a more barbed shape (Figure 1e). A larger grafted MCC particle with a barbed nature could denote that the amorphous areas of MCC experienced a different substitution level during the grafting process.^10,20^ This hypothesis agrees with the FTIR and NMR measurements, in which there was the emergence of some new bands in the amorphous areas of the modified MCCs.

#### 3.1.8 Contact Angle

Figure 1f shows the contact angle results of pristine MCC and different grafted micro-biosurfactants. Obviously, water has a much stronger interaction with pristine MCC surfaces compared to other modified MCC films. This denotes that the pristine MCC-based film typically shows a hydrophilic nature.^12,13^ Introducing TP into the MCC with no redox initiator compound (i.e., MCC/TP) offered a deteriorating effect on the surface hydrophobicity of pristine MCC. This may be associated with an alteration of the MCC structure as the interface nature among the blend phases could be weakened. In contrast, both free-radical grafting and dual-grafting reactions importantly increased the surface hydrophobicity of MCC. In this case, an increase in the water contact angle of the MCC film by a value of 20 and 33° was observed regarding MCC-*g*-TP and MCC-*g*-TP-*g*-PL, respectively. The strong interaction between the polar groups of MCC as the results of grafting reactions resulted in lesser hydrophilic sites on the film surface, which also offered a comparatively firmer structure.^13,20,23,24^

#### 3.1.9 Antioxidant Activity and Reducing Power

Figure 1g shows the antioxidant activity of pristine MCC and modified MCC conjugate variants. The lowest DPPH scavenging activity among all the assessed samples was detected for pristine MCC. To better elucidate the bioactivity properties of TP, we also measured the DPPH free radicals scavenging activity of the TP alone. The scavenging effect of TP was appreciably higher than the pristine MCC. This is perhaps not surprising as TP shows an actual apoptosis-inducing agent having a strong antioxidant character.^25^ Thus, the grafting of TP onto the MCC backbone reasonably induces a therapeutic application as an antioxidant compound. According to Figure 1g, the DPPH scavenging effect of MCC/TP (with no redox initiator compound) and MCC-*g*-TP was similar to that of the TP alone (*P* > 0.05). As the dual-grafting reaction progressed, the MCC-*g*-TP-*g*-PL more strongly captured the DPPH radicals in a dose-dependent manner compared to the free TP (*P* < 0.05). This result is likely due to an enlargement of the MCC-*g*-TP molecule after the ε-PL grafting reaction as a result of the polyphenol oxidation and oligomerization.^20^ This reaction contributes to a rise in the electron-donating groups, which makes the dual-grafted MCC show a more stable character compared to the TP alone. Therefore, the swelled MCC-*g*-TP-*g*-PL supramolecular more properly could quench the free radicals than the small molecule TP.

The reducing power activities of free TP compound, pure MCC, and grafted micro-biosurfactant are also presented in Figure 1g. All samples offered a high reducing power in a dose-dependent manner (*P* < 0.05), except pure MCC, which showed a poor reducing power property. In contrast to pure MCC, the MCC/TP and MCC-*g*-TP showed a stronger reducing power (*P* < 0.05), which was statistically similar to the free TP compound (*P* > 0.05). This is an expected outcome as TP has an active hydrogen-donating agent, making it a well-established antioxidant compound.^25^ The reducing power of MCC was increased more after the synthesis of the dual-grafted MCC conjugate (Figure 1g). This outcome suggests that the dual-grafting of TP and PL onto the MCC could enhance its antioxidant activity. The development of a stable system with a high-molecular-weight species is a result of the dual-grafting reaction, quenching efficiently the free radicals compared to free molecule TP. Thus, we successfully reinforced the MCC backbone by the development of MCC-*g*-TP-*g*-PL, which offers an efficient antioxidant activity.

#### 3.1.10 Antimicrobial Properties

A disk diffusion experiment was applied to determine the antimicrobial activity of the pristine MCC and modified MCCs (Figure 1g). The film discs of pristine MCC did not offer an inhibition area against any of the evaluated microorganisms after 24 and 48 h. Likewise, the MCC/TP and MCC-*g*-TP did not show an inhibitory effect against any of the evaluated microorganisms. In contrast, the film discs of MCC-*g*-TP-*g*-PL presented a great inhibitory behavior with a continuous inhibition effect after 24 and 48 h (*P* < 0.05). This observed antimicrobial property is strongly related to the ε-PL component,^26^ which was also according to the inhibitory impact of the free ε-PL component measured in this work (Figure 1g). The presence of a positive charge on the protonated guanidine group of ε-PL offers an effective antimicrobial activity. This positive charge disrupts the cell membranes of bacteria without triggering the cell lysis. However, it might also have an inhibitory impact on the other intracellular membranes leading to the bacteria lethality.^26^ Compared to *Salmonella enterica* and *Escherichia coli* O157:H7, the antimicrobial MCC-*g*-TP-*g*-PL films more inhibited the growth of *Listeria monocytogenes*. The obtained data specified a promising inhibitory effect of the dual-grafted MCC conjugate to improve product safety and industrial application.

### 3.2 Characterization of SPI-Based Pickering Emulsion Gels

#### 3.2.1 Structure of the Pickering Emulsion Gel

**Scheme 2:**
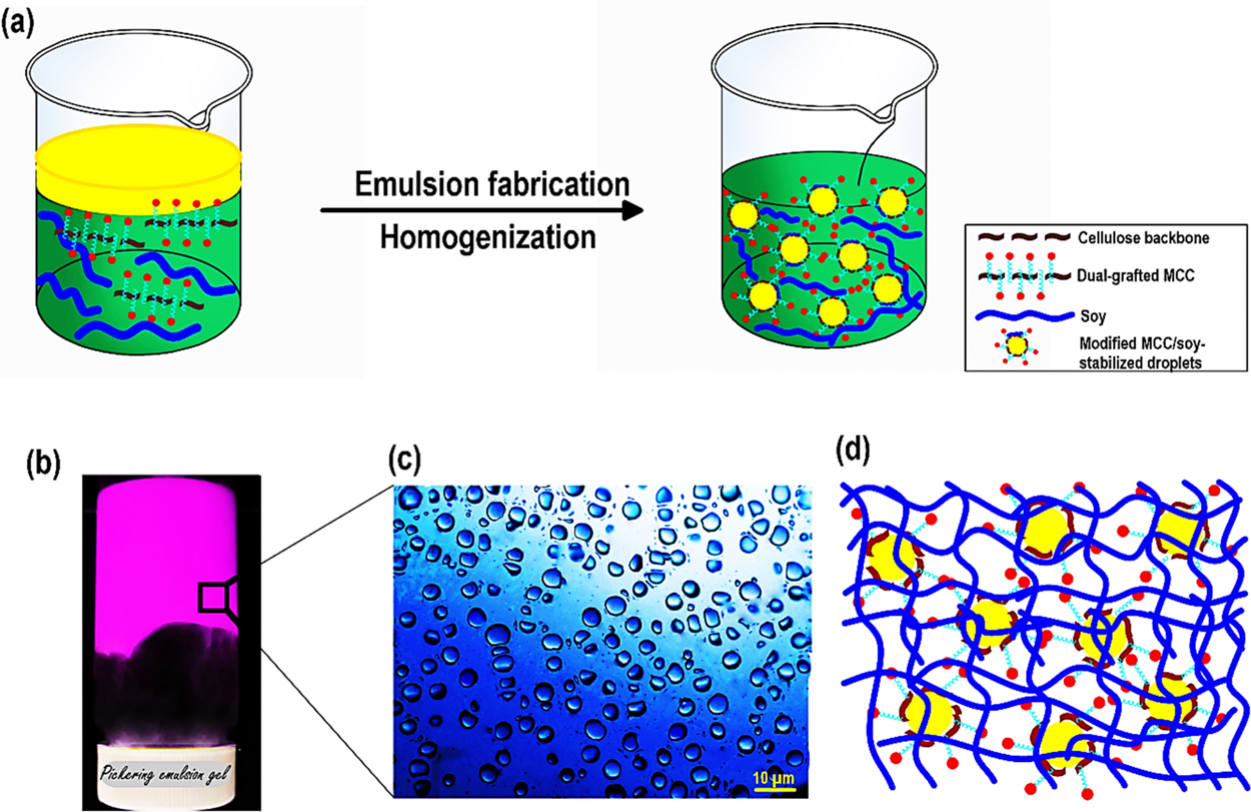
(a) Graphic Design (not to Scale) Concerning the Development of Dual-Grafted MCC/Soy-Stabilized Pickering Emulsion Gel; (b) the Visual Appearance of the Produced Pickering Emulsion Gel Kept in a Reversed Vessel; the Pickering Emulsion Gel was Obtained with 4 wt % Sunflower oil, 4.2 wt % MCC-*g*-TP-*g*-PL, and 25.0 wt % SPI and Stored under an Ambient Condition for 36 h; (c) the Optical Image of the Synthesized Pickering Emulsion Gel Including Some Large Droplets; and (d) Proposed Graphical Distribution of the Produced Pickering Emulsion Gel

Scheme 2 shows a theoretical graphic design of the method used to fabricate the antioxidative and antimicrobial SPI-based Pickering emulsion gels (Supporting Information, Section S.3). The SPI and grafted MCCs (both MCC-*g*-TP and MCC-*g*-TP-*g*-PL) comprise the continuous gel-like phase, which is exposed to the electrostatic repulsion due to their negatively charged residues (Supporting Information, Section S.6.6). As the optical image illustrated, the dual-grafted MCC/SPI-stabilized droplets are well dispersed in the system (Scheme 2). In this case, the developed multiphase blend system offers an effective way to compatibilize the hydrophobic phase using the amphiphilic grafted MCC or soy proteins.^13,20^ A little flocculation in the system may show the depletion mechanism of the non-adsorbed soy or modified MCC (Scheme 2). Thus, the produced Pickering emulsion gel can be considered a robust system, which simply integrated different components with variable surface energy patterns. This multiphase gel-like emulsion could be generalized to formulate other printable bio-based emulsions to manufacture the 3D printed architectures with certain functions, including antimicrobial, bio-absorbability, magnetic, and conductive (thermal/electric) agents.^6^

#### 3.2.2 Particle Diameter and PDI

**Figure 2:**
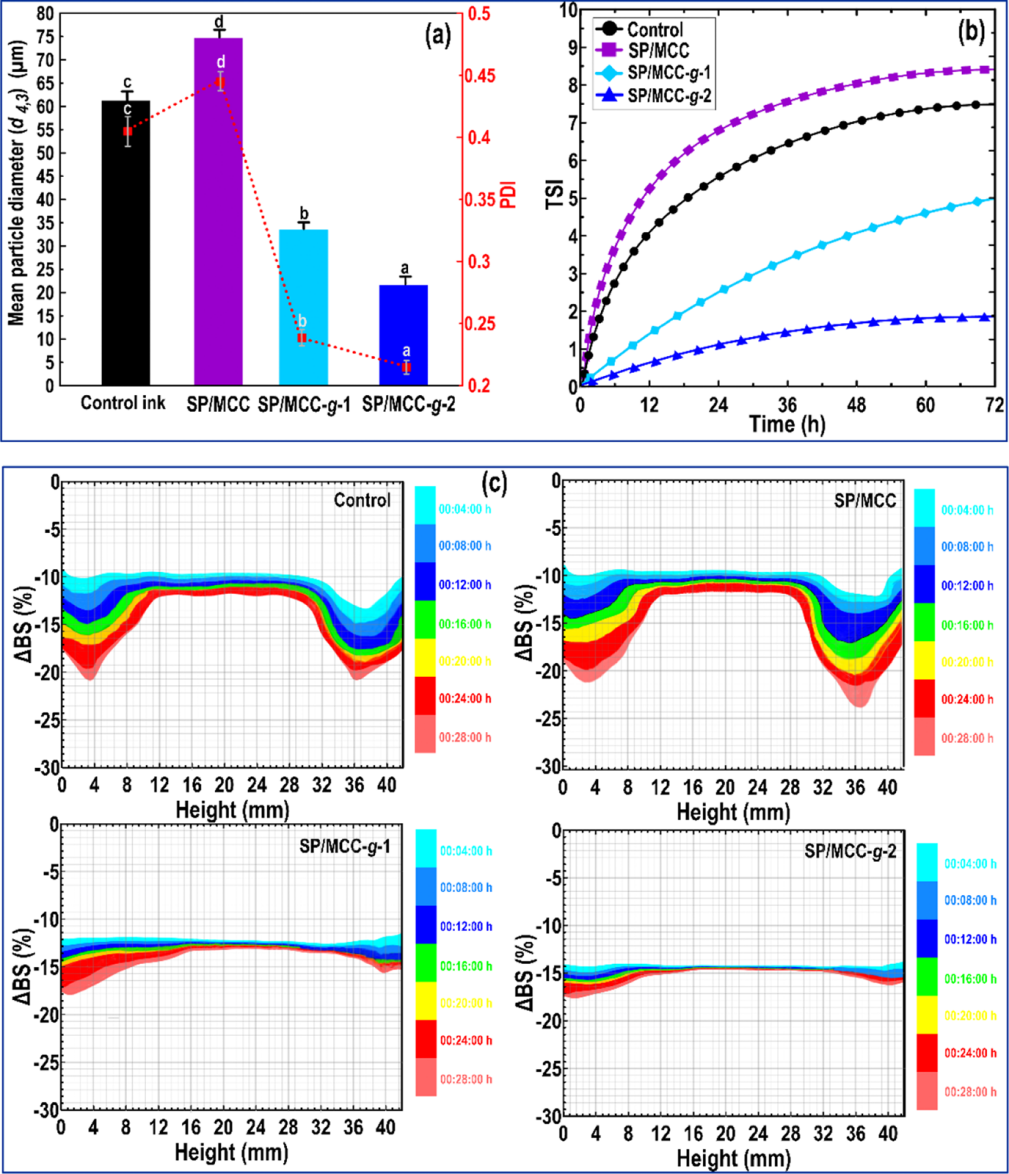
Characterization of SPI-based Pickering emulsion gels stabilized by pristine or modified MCCs. (a) (*d* 4,3) and PDI parameters, (b) TSI, and (c) (ΔBS) plots. Regarding (*d* 4,3) and PDI, the means in each column with various letters (a–d) are significantly different (*P* < 0.05) according to Duncan’s test.

The particle size and polydispersity index (PDI) are two paramount features to evaluate the stability of emulsions.^10^ Herein, the volume mean diameter (*d* 4,3) and PDI of control (SPI-based emulsion), SP/MCC, SP/MCC-*g*-1, and SP/MCC-*g*-2 inks upon 48 h storing are shown in Figure 2a. The initial volume mean diameter [(*d* 4,3) = 61 μm] and polydispersity index (PDI = 0.39) of the droplet-coated SPI (control ink) were comparatively large. This reveals that there are some flocculated oil droplets in the system with a non-uniform particle size distribution.^10,20,28^ Similarly, the addition of pristine MCC into the SPI-based emulsion (SP/MCC ink) promoted droplet aggregation as shown by data for the droplet size distribution (Figure 2a). The pristine MCC particles typically show a hydrophilic nature having a high electrostatic charge on their surface.^12^ Therefore, they cannot adsorb at the O/W interfaces, and thus offers poor emulsion stability.^13^ On the contrary, the partial replacement of oil by both MCC-*g*-TP (i.e., SP/MCC-*g*-1 ink) and MCC-*g*-TP-*g*-PL (i.e., SP/MCC-*g*-2 ink) developed a gel-like emulsion with a lower volume mean diameter as compared to the pristine MCC (i.e., SP/MCC ink) (Figure 2a). The grafted MCCs conjugated, being more hydrophobic (Figure 1f) with less charge density (Supporting Information, Section S.6.6), rationally tend to form the aggregated networks, offering the presence of small droplet sizes.^6,20^ Compared to SP/MCC-*g*-1 ink [(*d* 4,3) = 33.8 μm], the volume mean diameter of SP/MCC-*g*-2 ink was more reduced [(*d* 4,3) = 22.4 μm] possibly due to a more hydrophobic nature of MCC-*g*-TP-*g*-PL (Figure 1f). This leads to the conclusion that the dual-grafted MCC is capable of developing small droplets, and may adsorb quickly and induce a highly stable ink.^10,20^

Figure 2a also displays the PDI of different inks, which was shown to depend on the type of modified MCC conjugates. Compared to control ink (PDI = 0.39), the PDI of SP/MCC ink (PDI = 0.48) was significantly increased (*P* < 0.05). As the pristine MCC is a highly charged particle, it cannot adsorb on the surface of the oil droplets for providing the inter-droplet forces.^10,13^ However, the PDI of SPI-based ink was considerably reduced after the partial oil replacement by MCC-*g*-TP or MCC-*g*-TP-*g*-PL (*P* < 0.05). This effect may have been caused by an increase in the surface hydrophobicity of the grafted MCC micro-biosurfactants (Figure 1f), thus improving its surface activity.^20^ In this case, the PDI of SPI-based Pickering emulsions containing MCC-*g*-TP-*g*-PL (PDI = 0.225) was rather lower than that of containing MCC-*g*-TP (PDI = 0.251) (Figure 2a). As PDI is a measure of the particle size uniformity in a dispersion, a lower PDI shows a stable emulsion with a uniform particle size distribution.^10^ Therefore, the MCC-*g*-TP-*g*-PL endowed a stable Pickering emulsion gel with improved uniformity of the particle sizes in SPI-based Pickering emulsion gel.

#### 3.2.3 Determination of Pickering Emulsion Stability

The Turbiscan stability index (TSI) values of different emulsion systems were calculated and plotted as a function of time (Figure 2b). The partial oil replacement by pristine MCC (i.e., SP/MCC ink) produced an important increase in the TSI value compared to the control ink. This could be attributed to the oil droplets’ flocculation as a result of introducing pristine MCC, where the droplets could not be well-dispersed in the system.^10^ The adsorbed layer thickness of SPI and pristine MCC could be possibly insufficient to endow the stability through the steric repulsions. In contrast, the TSI parameter showed that both MCC-*g*-TP and MCC-*g*-TP-*g*-PL developed a stable SPI-based ink (Figure 2b). In this case, the Pickering emulsion gel formulated by MCC-*g*-TP-*g*-PL was more stable than that containing MCC-*g*-TP. This improved emulsion stability could be associated with an increase in the surface coverage of oil droplets due to the higher surface hydrophobicity of grafted MCC micro-biosurfactants. This exhibits an effective interfacial adhesion between the modified MCCs and the hydrophobic phase.^10,13^ Alternatively, the dual-grafting of TP and ε-PL onto the MCC enhanced the charge density and thickness of oil droplets. This improves the flocculation stability of droplets by increasing the electrostatic repulsion and steric between them.^20^

A vertical laser profiling stability analysis was used to further detect the stability of emulsion inks after 28 h storage at 25 °C, which performs according to the transmission (Δ*T*) and delta-backscattering (ΔBS) patterns (Figure 2c). The *X*-axis denotes the height of the tested bottle, and *Y*-axis shows the percent change of BS relative to the initial state. The colors of the plot are related to the different times on the second *Y*-axis.^10^ The ΔBS of control ink was decreased overall with time, signifying an incremental rise in the particle size caused by the flocculation or coalescence phenomena (Figure 2c). This result also agrees well with the previously obtained TSI data (Figure 2b). Additionally, there is an increase in the peak widths of control ink in both the bottom and top of the evaluated bottle as a function of time. The migration of larger droplets from the bottom to the top of the bottle was likely performed because of a density difference between water and oil (creaming phenomenon).^10^ Compared to control ink, the ΔBS of the bottom and the top layer of SP/MCC ink was more decreased over time as well, denoting a significant phase separation. On the contrary, the BS of inks prepared with both grafted and dual-grafted MCCs presented slight variation in the lower-middle-upper part (20–35 mm), with a little reduction at the top (40–42 mm), and a small rise at the bottom (0–5 mm) of the evaluated bottle (Figure 2c). Moreover, the ΔBS profile at the bottom of the bottle was slowly decreased for these Pickering emulsions. The smaller particle size of the oil droplets rationally decreases the creaming rate.^10^ The modified MCC conjugates could effectively coat the oil droplets and rigidify the interfacial film of newly formed emulsion droplets and therefore inhibiting the incidence of the coalescence phenomenon.^4^ Among all the inks studied here, a slight alteration in the visual appearance of the ink prepared with the dual-grafted MCC conjugate was noticed after 28 h of storage. Therefore, it could be a promising method to predict the long-term physical stability of SP/MCC-*g*-2 ink through the short-term vertical laser profiling assay.

#### 3.2.4 Microstructure of Pickering Emulsion Gels

**Figure 3:**
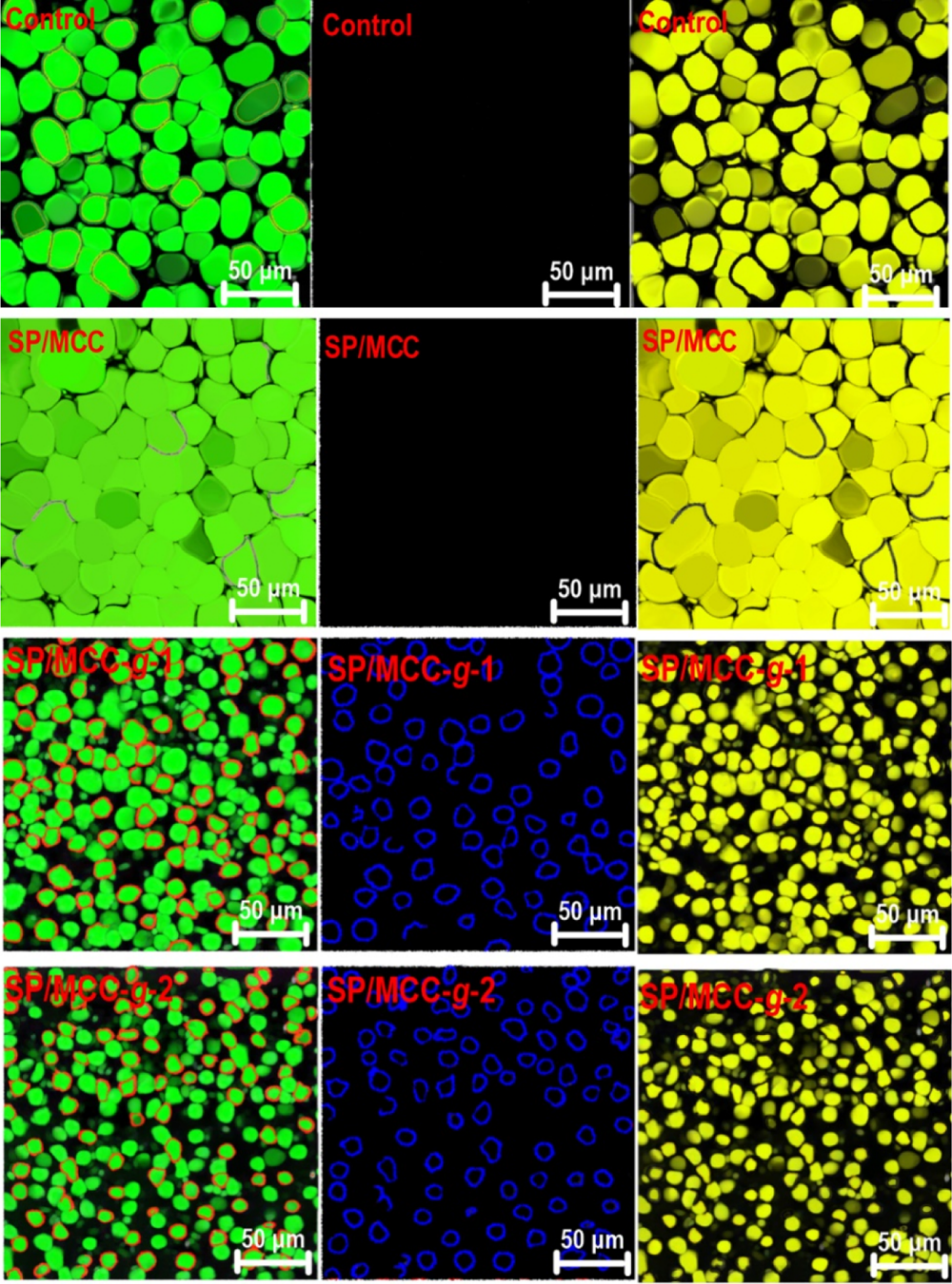
CLSM micrographs of soy-based Pickering emulsion gels stabilized by pristine or grafted MCCs. The right column denotes the oil phase. The middle column specifies modified MCC/soy-stained blue. The left column presents overlapping images. For better clarification, the obtained colors were slightly edited.

The morphology and microstructure of ink variants were evaluated by confocal laser scanning microscopy (CLSM) (Supporting Information, Section S.4.3). The CLSM offers desired quality with a high-resolution image of the internal structure and interfacial framework. As Figure 3 depicted, the oil droplets in control and SP/MCC inks seemed to be the largest and there were small spaces between them. This instability could be due to the lack of a surface-active compound (such as the grafted MCC conjugates) being present at the developed O/W interface for the surface coverage.^13,28^ Thus, the flocculation and coalescence of the oil droplets were likely developed in the system. Compared to control and SP/MCC inks, the oil droplets were more uniformly dispersed in the continuous phase regarding the inks prepared with the grafted MCC micro-biosurfactants, especially SP/MCC-*g*-2, showing a smaller size (Figure 3). This result also agrees well with the light scattering data (Figure 2a). Figure 3 shows that there are three fluorescence images for each Pickering emulsion gel. This includes the oil phase (right column), soy protein/MCCs (middle column), and both oil and soy protein/MCCs stained, that is, overlapping images obtained by exciting Nile Red and Nile Blue A (left column). The oil droplets in the overlapping fluorescence images comprised the interior green phase, while the red color as a shell around the droplets was formed by soy protein/MCCs. This suggests that an O/W-type emulsion was effectively deloped^28^ with no noticeable droplet coalescence in the system. Apparently, the densely packed layers of grafted MCCs (along with soy proteins) on the surface of spherical oil droplets provided enhanced system stability. The formed interfacial layers allowed a solid barrier for the emulsion gels, reinforcing the physical stability against flocculation, coalescence, and Ostwald ripening.^29^ It should be noted that a more solid on-adsorbed MCC film layer (and also soy proteins) seems to be developed in the presence of the dual-grafted MCC. This provided the depletion flocculation by an osmotic pressure gradient within the continuous phase surrounding the droplets, where the following aggregation led to the stronger interaction between droplets.^13^ Compared to SP/MCC ink, the droplets in SP/MCC-*g*-1 and SP/MCC-*g*-2 ink showed a smaller size, which was more uniformly dispersed in the continuous phase. These results are in accordance with those of particle size and TSI measurements. Therefore, the grafted MCC conjugates offered better stabilization performance, where their corresponded inks showed smaller droplet sizes with a nonaggregated bound droplet that were homogeneously distributed throughout the continuous phase.

#### 3.2.5 Dynamic Interfacial Pressure and Interfacial Viscoelasticity Experiments

**Figure 4:**
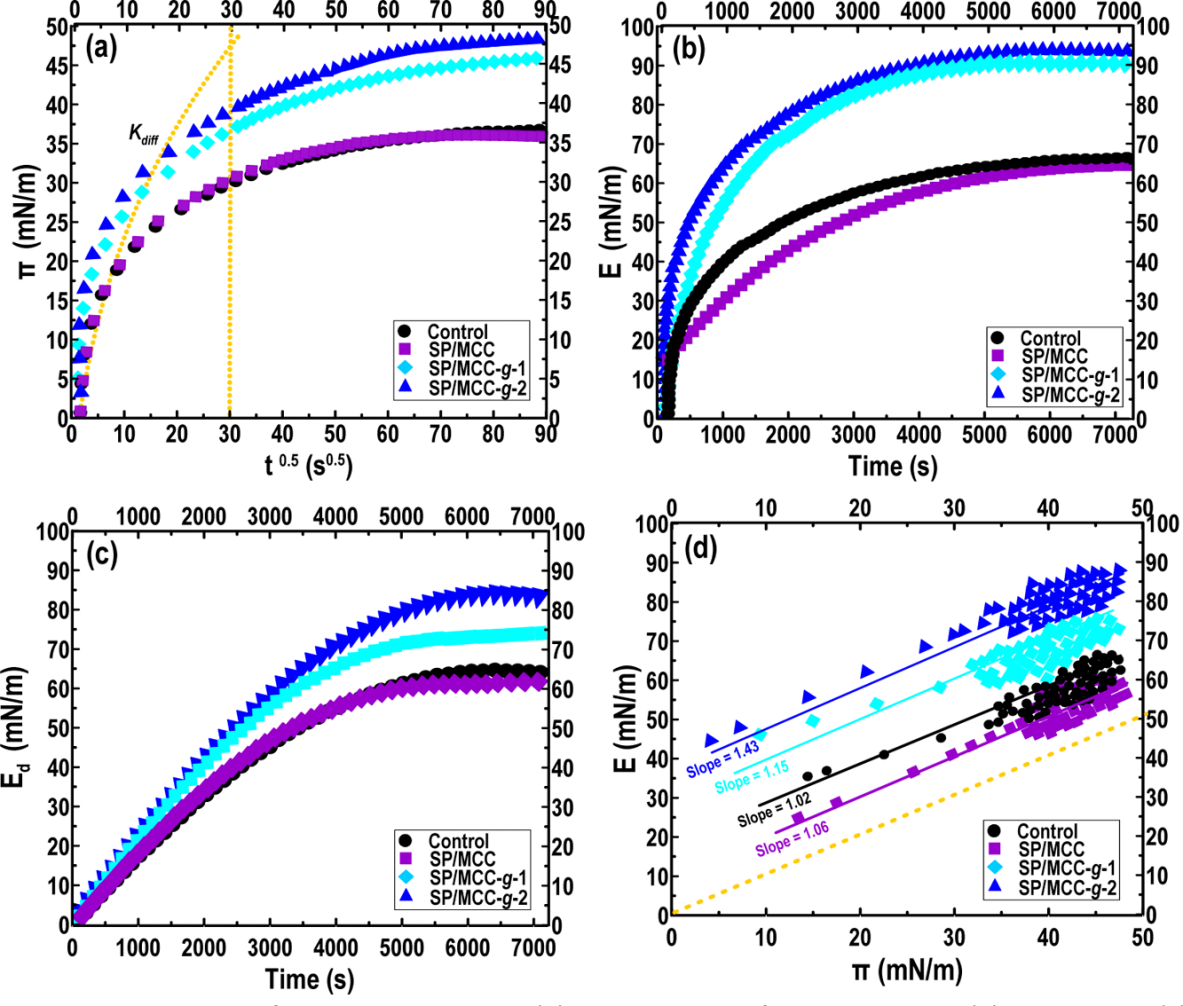
Time-dependence of adsorption kinetics (a), as well as surface dilatational (b) and elastic (c) moduli regarding adsorption of soy protein alone and soy protein containing MCCs at the O/W interface. Surface dilatational modulus (*E*) as a function of surface pressure (π) for soy protein alone and soy protein containing MCCs at the O/W interface (d).

Figure 4a shows the interfacial surface pressure (π) as a function of time for control or SPI/MCCs inks at the O/W interface. Regardless of sample type, the π increased quickly within the first 20 min. With further increase in time, the value of π levels off as the surface becomes saturated with SPI or SPI containing MCCs at the O/W interface. Compared to control and SP/MCC inks, the plateau π values of SP/MCC-*g*-1 and SP/MCC-*g*-2 inks were faster raised within the first 20 min, in which the π value showed the highest value regarding SP/MCC-*g*-2 (Figure 4a). The diffusion rate (*K*_diff_) was also calculated from the π versus *t* 0.5 (≤30 s^0.5^), and the obtained fitting data are given in Table S2 (Supporting Information). The SP/MCC-*g*-1 and SP/MCC-*g*-2 inks showed a lower *K*_diff_ than those of control and SP/MCC inks (*P* < 0.05), suggesting that the grafted MCC micro-biosurfactant had the desired impact on the diffusion–adsorption of SPI particles. This could be attributed to the formation of aggregated particles and/or aggregated networks, slowing down the adsorption rate of the solid particles at the O/W interface. In accordance with these results, the surface dilatational (Figure 4b) and dilatational elastic (Figure 4c) moduli of SP/MCC-*g*-1 and SP/MCC-*g*-2 were noticeably higher than those of SPI alone or those of SP/MCC inks. The slope of the *E*–π plot was also measured (Figure 4d), which represents the adsorption magnitude of the colloidal particles at the O/W interface. All the slopes were higher than 1.0, with the slopes of SP/MCC-*g*-1 and SP/MCC-*g*-2 inks being higher in comparison with SPI alone or with SP/MCC inks. This indicates a nonideal adsorption behavior at the O/W interface. The obtained data verified that the interactions of SPI with both grafted or dual-grafted MCC conjugates could improve the strength of adsorbed layers and enhance the interfacial viscoelasticity.

The interfacial adsorption behaviors of SPI-containing MCCs, as well as Δ*f* and Δ*D* from the fifth harmonic as a function of time, were evaluated (Supporting Information, Figure S7), which showed the mass deposition of both SPI and MCC particles onto the oil-coated surfaces. Regarding SP/MCC-*g*-1 and SP/MCC-*g*-2 inks, the negative Δ*f* was reduced abruptly after 10 min (Supporting Information, Figure S7a). This could be related to a higher viscosity (see Section 3.2.6) and/or the density of particle dispersion. The rigidity and viscoelasticity of the adsorbed film were strongly affected the Δ*D*. A thin and rigid layer shows a negligible impact on the Δ*D* (< 1 × 10^–6^ Hz), while a thick and flexible film structure causes a greater Δ*D*. In the current work, a rapid positive increase of Δ*D* for SP/MCC-*g*-1 and SP/MCC-*g*-2 inks was observed during the 10 min period, which also showed a comparatively higher value compared to control and SP/MCC inks (Supporting Information, Figure S7b). Once again, this result demonstrates the development of thick and flexible adsorbed SPI and modified MCC layers.

According to the measured Δ*m* (Supporting Information, Figure S7c), the adsorbed surface coverage of SP/MCC-*g*-1 and SP/MCC-*g*-2 were also increased more in contrast to control and SP/MCC ink samples. This in turn could manifest itself as a change in the viscoelastic modulus and interfacial pressure. Compared to the adsorption of SPI and/or MCC-*g*-TP and MCC-*g*-TP-*g*-PL onto the oil-coated surfaces, the SP/MCC-*g*-1 and SP/MCC-*g*-2 developed a larger adsorbed amount, thus forming a thicker and indeed more flexible film. The adsorption behavior data showed that the SPI and grafted MCC-*g*-TP and MCC-*g*-TP-*g*-PL contributed to the development of an interfacial film, promoting the formation of the thick and flexible layers.^27,30−32^

#### 3.2.6 Steady Shear Flow Behavior

**Figure 5:**
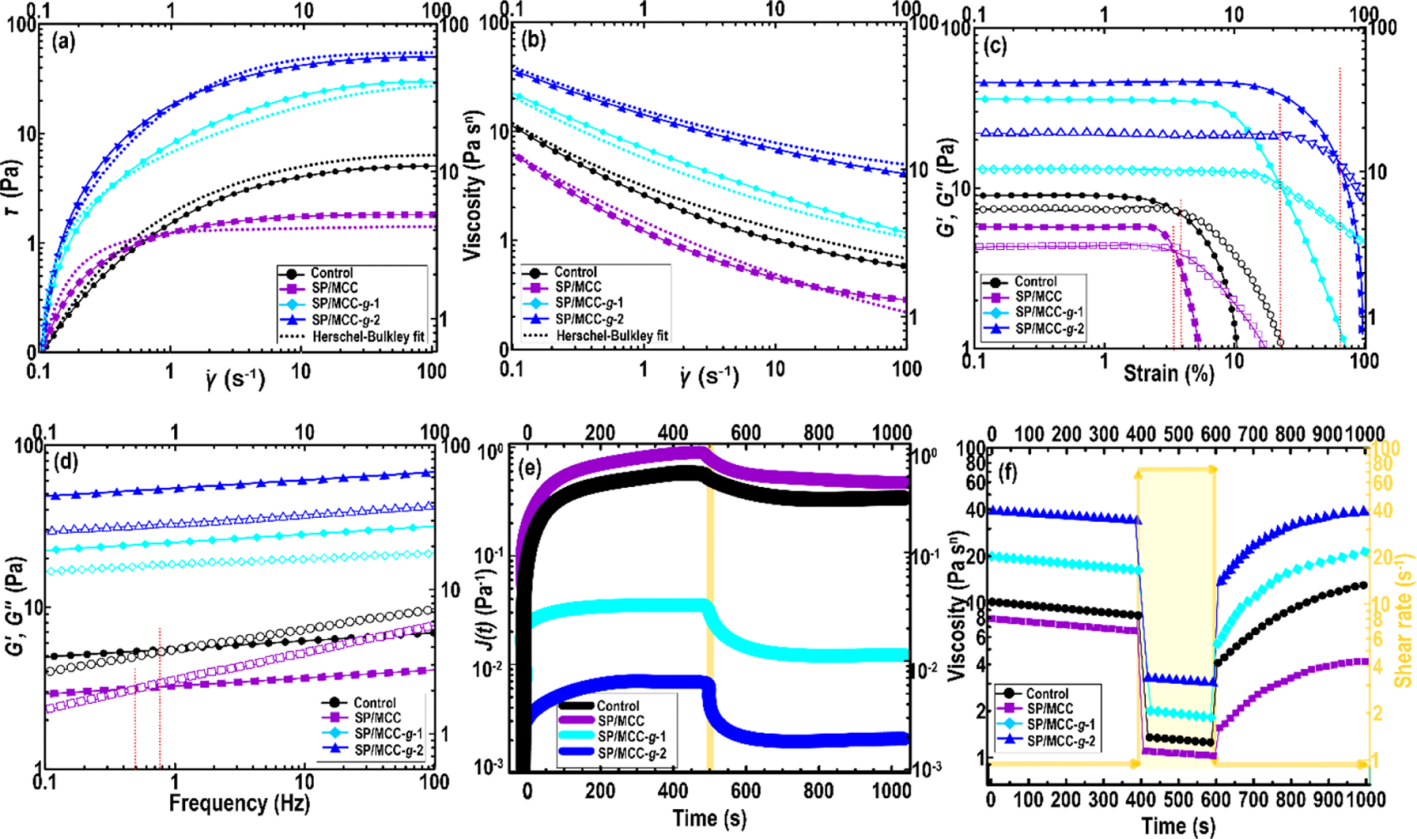
(a) Shear stress and (b) viscosity dependence on the shear rate of SPI-based Pickering emulsion gels. (c) Strain sweep and (d) frequency sweep curves of Pickering emulsion gels, in which solid symbols denote *G*′ and open symbols specify *G*″. (e) Creep and creep-recovery plots and (f) the 3-ITT of Pickering emulsion gels.

The shear rate dependency of the stress (Figure 5a) or apparent viscosity (Figure 5b) of different Pickering emulsion gels was investigated. There was a characteristic non-Newtonian pseudoplastic behavior regarding all emulsions at a shear rate of 0.1–100 s^–1^.^33^ From Figure 5b, the viscosity of all inks offered a high value at the low shear rate (<1 s^–1^) and after that reduced with the increase in shear rate. Interestingly, the apparent viscosity of control SPI-based ink tended to decrease after oil replacement by pristine MCC (i.e., SP/MCC). This could be due to a high charge density (Supporting Information, Section S.6.6) and poor emulsion stability (Section 3.2.3) of pristine MCC. However, the viscosity of SPI-based ink was increased after the addition of modified MCCs. Both grafted MCC conjugates with a surface-active property connected the SPI-coated oil droplets together,^20^ forming an aggregated network and increasing the viscosity of the system.

In the 3D printing process, a shear-thinning behavior causes the ink to be extruded out easily through the nozzle tip using a rational extrusion shearing force.^2,4,13,28^ The flow behavior index of control emulsion, obtained from the Herschel–Bulkley model fit (Supporting Information, Table S3), was determined to be 0.91, showing a characteristic weak associative interaction. This represents that a poor droplet network structure was likely developed in the SPI-based emulsion as reported earlier.^4,10^ Compared to control ink, there was an increase in the flow behavior index of SP/MCC ink (Supporting Information, Table S3), denoting still the presence of a weak shear-thinning behavior. In contrast, the oil replacement by both grafted MCCs led to a significantly lower flow behavior index (*P* < 0.05). This behavior could be explicated by breaking the aggregated droplets into smaller clusters during shearing, offering more shear-thinning character.^10,20^

The yield stress is also a critical rheological parameter in defining a suitable ink for extrusion-based printing, influencing printability and shape fidelity.^2^ According to the flow behavior results (Supporting Information, Table S3), the unmodified MCC reduced the yield stress value of the SPI-based emulsion. The oil replacement by MCC-*g*-TP or MCC-*g*-TP-*g*-PL caused a notable increase in the yield stress. The obtained result is possibly related to the fact that the surface-active MCCs could develop an aggregated network because of their higher hydrophobicity (see Figure 1f), thus improving the elasticity of the system. High yield stress would be necessary during 3D printing purposes; otherwise, the layer of 3D printed structures might suffer an intolerable collapse or deformation during the printing process.^2,4,20,28^

#### 3.2.7 Strain Sweep

Figure 5c presents the results of amplitude sweep in the terms of storage (*G*′) and loss (*G*″) moduli. A linear viscoelastic regime (LVR) was detected at a small strain amplitude (0.1 < γ < 1). In this case, the *G*′ values were greater than those of *G*″ for all inks.^33^ This denotes that there was the existence of an elastic-like behavior, signifying that under a nondestructive condition, the elasticity dominates the viscosity.^4^ As Figure 5c visualized, beyond the LVR (entering the non-linear area), both *G*′ (γ) or *G*″ (γ) moduli decreased. Compared to control ink, the *G*′ (γ) values of SP/MCC ink were lower, which was probably due to the lower total effective volume fraction of the SP/MCC system offered by pristine MCC. This shows the development of less structured and connected networks.^10,13^ In contrast, SP/MCC-*g*-1 and SP/MCC-*g*-2 inks showed a higher value of *G*′ (γ) and *G*″ (γ), which clearly denotes that a more structured and stable system was fabricated. In agreement with this behavior, these inks presented a higher static yield stress among all the evaluated inks (Supporting Information, Table S3). The obtained results further support the fact that the addition of grafted MCCs led to an increase in the effective size of the aggregated oil droplet clusters with the formation of aggregated networks on the structure of SPI-based emulsion.^16,20^

#### 3.2.8 Frequency Sweep

To evaluate the dependence of the viscoelastic parameters of Pickering emulsion gels on the angular frequency (ω), a frequency sweep test was performed (Figure 5d). A typical gel-like behavior was observed in all inks as the *G*′ (ω) values were higher than those of *G*″ (ω) at the low frequency (<1 Hz). Besides, the viscoelastic parameters displayed a slightly linear increase as a function of angular frequency (Figure 5d). At the initial area of the frequency sweep test (0.1–1 Hz), the *G*′ (ω) of control or SP/MCC inks prevailed over *G*″ (ω). However, at the higher angular frequency (>3 Hz), the *G*″ (ω) plots crossed over with those of *G*′ (ω). This indicates a maximum energy dissipation, representing that the viscoelastic solid-like property changed to a viscoelastic liquid behavior. The frequency sweep results also showed that the pristine MCC weakened the gel-like structure of the SPI-based emulsion.^10^ In this case, the oil replacement by pristine MCC led to an appreciable decrease in the *G*′ (ω), subsequently resulting in the development of a less structured system. Compared to the control ink, the viscoelastic moduli of SP/MCC-*g*-1 and SP/MCC-*g*-2 inks showed higher *G*′ (ω) or *G*″ (ω) values, proposing the development of a more structured system. Moreover, their plots exhibited a slightly linear rise as a function of frequency; however, their values showed *G*′ (ω) > *G*″ (ω) throughout the frequency sweep measurement with no detected cross-over point. The frequency sweep data, thus, confirm the results of steady shear flow behavior and strain sweep assays reported earlier. The important enhancement in the elastic behavior of the Pickering emulsion gels was possibly associated with a greater density of crosslinks between the droplets, provided by MCC-*g*-TP or MCC-*g*-TP-*g*-PL.^13,20^

#### 3.2.9 Creep and Creep-Recovery Measurements

The maximum creep compliance level of control SPI-based ink was measured at about 0.7 Pa^–1^ (Figure 5e). The incorporation of unmodified MCC into the SPI-based ink increased the maximum creep compliance to around 1.1 Pa^–1^. This signifies a decrease in the elasticity of the ink system due to the lack of development of a structured emulsion. However, the maximum creep compliances of SP/MCC-*g*-1 (*J*(*t*) = 0.03 Pa^–1^) and SP/MCC-*g*-2 (*J*(*t*) = 0.007 Pa^–1^) were 23.3- and 100-fold lower compared to control, respectively. Thus, the grafted or dual-grafted MCC conjugates effectively enhanced the elastic portion of the viscoelastic response. This could be related to the fact that the modified MCCs are prone to form an aggregated network owing to a high hydrophobicity character of MCC-*g*-TP or MCC-*g*-TP-*g*-PL.^10,20^

The level of declining material deformation after the elimination of applied stress in the creep evaluation denotes a creep-recovery phase. A higher elasticity and a solid-like structure reasonably show a higher relative recovery percentage.^13^ According to the recovery phase results, the recovery percentage of control SPI-based ink was measured at about 44%, showing the presence of a less reversible network. Similarly, SP/MCC ink presented a weak elasticity and an unstable structure with a recovery percentage of ∼37%. In contrast, the recovery percentages of SP/MCC-*g*-1 and SP/MCC-*g*-2 inks were detected to be ∼74 and ∼80%, respectively, which effectively recovered their original structures. This suggests that the structure of SPI-based ink was considerably reinforced as affected by both grafted MCCs.^20^

#### 3.2.10 Three Interval Thixotropy Test (3ITT)

The 3ITT includes a three-interval time-dependent thixotropic process to determine the degree of reconstruction of a molecular assembly after a shear-induced interruption. In this test, (i) a constant amplitude or frequency inside the LVR is performed to measure a reference state of the ink with no microstructure disruption, (ii) which is followed by a second interval, in which the ink’s microstructure is destroyed by a high amplitude or frequency. (iii) The third interval is similar assay as the first phase, which determines the reversible reconstruction of a molecular structure. Figure 5f depicts the viscosity dependence on applied time and deformation rates of different inks, performed by 3ITT. As expected, the viscosity of SP/MCC ink in the first time interval was lower than control ink, which agrees well with the reported results of steady shear flow behavior (Section 3.2.6). As the pristine MCC has a typical hydrophilic nature^10,12,13^ with a larger electrostatic charge,^20^ it lacks to be located at the formed O/W interface for surface coverage. Compared to control and SP/MCC inks, the Pickering emulsions formulated by MCC-*g*-TP or MCC-*g*-TP-*g*-PL offered a greater viscosity profile (Figure 5f). This indicates that the formation of a structured emulsion on account of a higher hydrophobicity of grafted MCCs.^5,20^

In the second phase (with a steady shear rate of 80 s^–1^), the interconnected structures of the inks were disrupted, resulting in a huge decrease in viscosity. Concerning the third stage, the viscosity of control and SP/MCC inks was much lower than that of the first stage, which could be attributed to irreversible molecular structure damage.^13,28^ A less structured gel-like matrix of these emulsions with a weakly connected network offered an inferior mechanical strength, showing an irreversible restructuration.^5,20^ This result is according to the steady and oscillatory rheological assays, in which an unstructured emulsion endowed a weak gel-like property. Compared to control and SP/MCC inks, an appreciably stronger structure of SP/MCC-*g*-1 and SP/MCC-*g*-2 emulsions was inferred from an outstanding structural recovery (Figure 5f), where the biopolymeric chains were sufficiently reordered as the molecular structure came to the new equilibrium phase.^10^ This follows the expected behavior of the Pickering emulsion gels with a strengthened structure, which offered the system resistance to the rapid strain. This result also agrees well with the reported creep-recovery measurement. In conclusion, the 3ITT proposed that the Pickering emulsion gels containing both MCC-*g*-TP and MCC-*g*-TP-*g*-PL offered a reversible restructuration of their initial network matrix, affecting the elastic or viscous components of viscoelastic response in the system.

### 3.3 Characterization of 3D Printed Architectures

#### 3.3.1 Printing Quality

**Figure 6:**
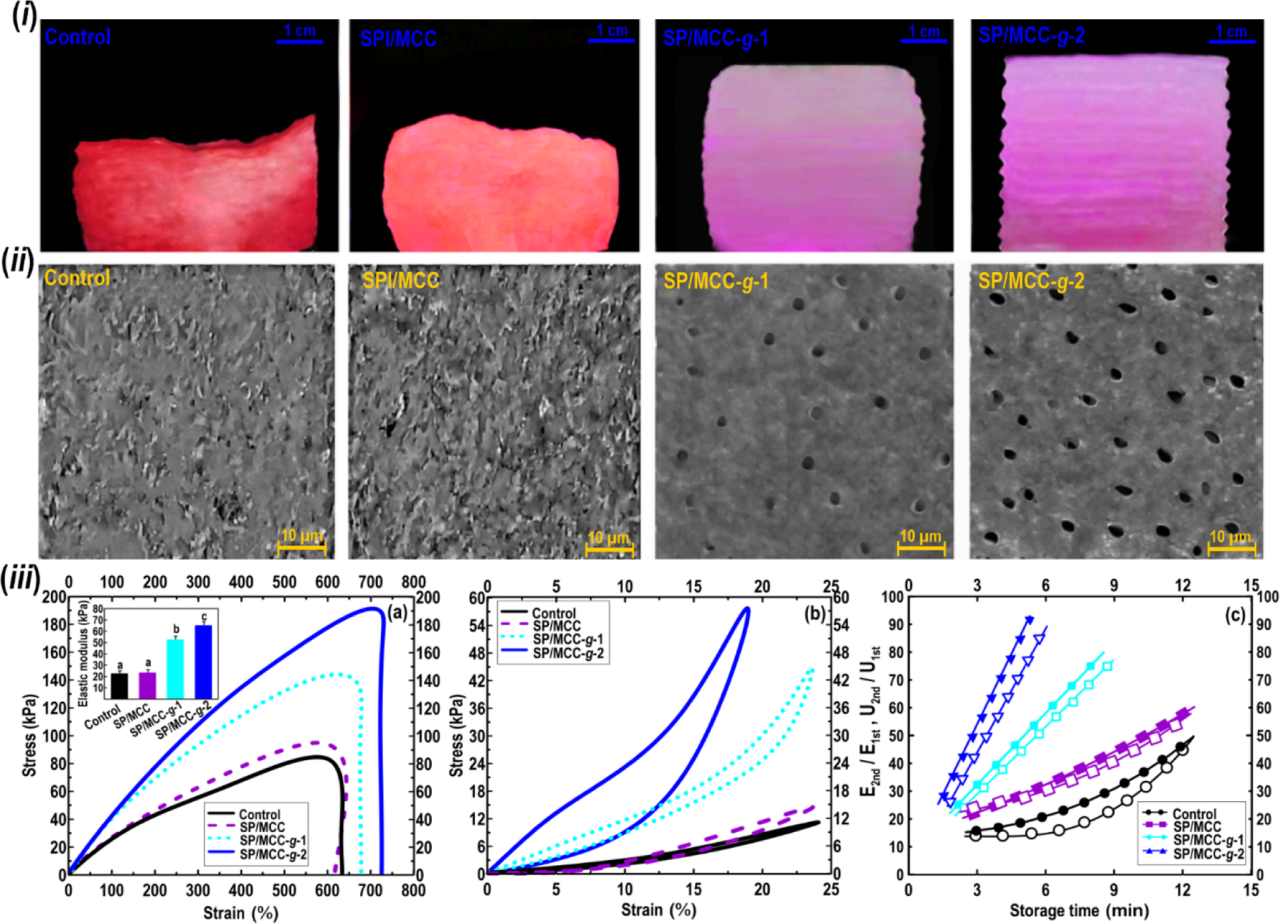
(i) Printing quality images of various 3D printed architectures. (ii): VP-SEM photomicrograph of 3D printed structure variants. (iii) Stress–strain curves of different 3D printing architectures (a). Curves of loading–unloading cycles (b). Proportion of elastic modulus and energy dissipation (*U*) upon the second loading–unloading cycle to those during the first one for the relaxed and notched samples kept at 90 °C plotted against different storing times (c).

Printing quality is an imperative prerequisite to determine the success of 3D printing process. It can be generally evaluated according to the appearance of 3D printing objects, instead of being quantified systematically.^2,20,34^ The emulsion ink samples were loaded with different formulations and layer-by-layer printed through an extrusion-based 3D printer. The control SPI-based ink spread on a tray immediately once deposition, where the layers were relatively unsupportable (Figure 6, row i). Thus, it offered an unstructured network system, leading to a poor precision geometry with a low spatial resolution. Likewise, the SP/MCC ink could not develop a suitable geometry after the printing process. In this case, its corresponding ink showed low yield stress (i.e., the minimum force necessary for the extrusion 3D printing), poor elastic property, and a modest thixotropic feature. This offered the manufactured 3D printed architecture with an inferior shape retention ability and unstable structural property.^34,35^ In contrast, introducing both grafted MCCs to the SPI-based emulsion allowed the formation of well-defined 3D printed objects, which were self-supported because of appropriate yield stress and viscoelastic modulus (Figure 6, row i). In these cases, the geometrical structures of the 3D objects were also appropriately maintained with no obvious distortion, cracking, and remarkable volume shrinkage. Compared to the 3D printed SP/MCC-*g*-1 object, the printed SP/MCC-*g*-2 showed a better printing quality and dimensional stability. This leads to the 3D printed SP/MCC-*g*-2 object taking desirable durability supporting the subsequently deposited layers, thus resulting in a high resolution and shape-fidelity.

#### 3.3.2 Morphological Observation of 3D Printed Architectures

The VP-SEM microstructures of 3D printed SPI-based structures fabricated by various types of MCC are shown in Figure 6 (row ii). A rugged and uneven microstructure with some obvious agglomerated fragments was observed regarding 3D printed control. In addition, no apparent pore structure was detected within its matrix. As Figure 6 (row ii) also depicted, the addition of unmodified MCC into SPI following 3D printing harmed its microstructural property, where the surface of 3D printed SP/MCC presented some irregularity with clear aggregated micro-sized pieces. Alternatively, a partial presence of a small number of pores was found in the 3D printed SP/MCC-*g*-1, which showed a small size and a spherical form. Similarly, the VP-SEM image of 3D printing SP/MCC-*g*-2 showed the attendance of a high level of pore structures, which offered a smaller size and a more regularly distributed pattern within the matrix. The microstructure results could explain the use of modified MCCs in the SPI-based emulsion led to a difference in its morphological feature as a result of better rheological and structural stability of 3D architectures.^2,6,10,20^

#### 3.3.3 Mechanical Strength of 3D Printed Constructs

The mechanical strength and toughening mechanism of the SPI-based printed samples stabilized by pristine or grafted MCCs were explored.^36^ The mechanical data showed that the elastic modulus, *E* of the SPI-based printed structure was enhanced after the incorporation of modified MCCs (Figure 6a, row iii). This suggests that the mechanical strength of SPI-based printed structures comes from the contribution of both MCC-*g*-TP and MCC-*g*-TP-*g*-PL to the structuring of Pickering emulsion gels. In this case, 3D printed SP/MCC-*g*-2 (i.e., the SPI emulsion containing MCC-*g*-TP-*g*-PL) showed a higher toughness compared to SP/MCC-*g*-1, where the highest fracture energy was obtained.

Throughout the loading and unloading measurements, the fracture mechanism and the toughening phenomenon of 3D printed objects were further evaluated. At a small tensile strain (below the yield strain of 3D printed structures), 3D printed SP/MCC-*g*-2 object displayed a noticeable degree of hysteresis and maintained an important level of enduring deformation upon unloading. Similarly, 3D printed SP/MCC-*g*-1 exhibited a characteristic hysteresis, while a slight hysteresis was detected for control and SP/MCC (Figure 6b, row iii).

To assess the breaking strength of 3D printed constructs, we also determined the proportions of elastic modulus, *E*_2nd_, or fracture energy, Γ_2nd_, in the second loading–unloading phase relative to their values in the first loading–unloading phase. Regardless of sample type, there was a notable reduction in the *E*_2nd_/*E*_1st_ and Γ_2nd_/Γ_1st_ with an increase of strain in the first loading–unloading cycle. This indicates that the elastically effective 3D structure was disrupted with the increase in the level of the extension (Supporting Information, Figure S8). In accordance with the earlier outcomes that the *E* magnitudes of printed SP/MCC-*g*-1 and SP/MCC-*g*-2 were greater than the elastic moduli of control and SP/MCC printed objects, it could be assumed that these printed structures are not only composed of aggregated networks but also consist of some interactions including hydrogen bonding between SPI and the grafted MCCs.^36^ This deduction if true has a positive impact on superior viscosity, higher viscoelasticity, and excellent thixotropic features of the SP/MCC-*g*-1 and SP/MCC-*g*-2 inks.

The recoverability of a notched 3D printed sample was further investigated (Figure 6c, row iii). Regarding 3D printed SP/MCC-*g*-1 and SP/MCC-*g*-2, the *E* parameter, and energy dissipation (*U*) were recovered to approximately 90 and 75%, respectively. This demonstrates that these 3D printed samples presented a desired recoverable matrix, which also agreed well with the creep-recovery test (Section 3.2.9) or the 3ITT (3.2.10). It was established that the *U* parameter in a multisystem sample (such as SPI and grafted MCCs studied here) is strongly associated with a physically crosslinked network, where the dissipated energy of such a system can be effectively recovered after relaxation.^36^

## 4 Conclusions

Pickering emulsion gels have been widely used as printable ink for biomedical, bioengineering, and food 3D printing. Inspired by a sustainable 3D printed therapeutic object, MCC was selected to manufacture a low-fat soy-based emulsion gel. However, owing to its poor physical stability, pristine MCC has rarely been employed for the stabilization of emulsion systems. In the present work, a multifunctional dual-grafted MCC was applied to stabilize a bioactive soy-based Pickering emulsion for application in the 3D printing process. We hypothesized that the grafting of the TPs and ε-PL onto the MCC backbone enhanced its emulsification properties because of the development of the Schiff-base reaction and/or Michael addition. The application of dual-grafted MCCs led to the production of a soy-based emulsion with smaller droplets and a uniform particle size distribution. Long-term emulsion stability was also obtained after the addition of modified MCCs. Both the advantageous features, that is, antimicrobial and antioxidant activities, of ε-PL and TPs appeared on the Pickering emulsion gel (and also 3D printed objects). The soy protein-based Pickering emulsion gel could be also effectively printed via an extrusion-based printer to fabricate a highly porous structure. The printing quality results demonstrated that the Pickering ink samples that included modified MCCs, had substantially enhanced the resolution of the deposited layers and offer a well-defined geometry. This study can look promising, from fundamental and practical attitudes, regarding the utilization of a therapeutic Pickering emulsion gel to manufacture an efficient 3D printed reduced-fat structure, broadening the application of a modified micro-biosurfactant in biomedical, bioengineering, and food 3D printing.

## Supporting Information Available

Materials; preparation and characterization of grafted MCC conjugates; preparation of soy protein-based Pickering emulsion; characterization of soy protein-based ink; characterization of 3D printed objects; results and discussion; freeze-dried pristine MCC powder, redispersed pristine MCC in water, and MCC redispersed in toluene; FTIR spectra; SEM image; DPPH scavenging activity; antimicrobial activity; stress sweep test; Δf and ΔD from the fifth harmonic as a function of time; Γ_2nd_/Γ_1st_ and E_2nd_/E_1st_ as a function of the strain of first loading in different printed objects; printing settings; diffusion coefficients; and obtained viscosity, flow behavior index, and yield stress (PDF)
