# Supplementary material for: Dual-Grafting of Microcrystalline Cellulose by Tea Polyphenols and Cationic ε-Polylysine to Tailor a Structured Antimicrobial Soy-Based Emulsion for 3D Printing
Source: ACS Appl Mater Interfaces. 2022 Apr 27;14(18):21392–405. doi: 10.1021/acsami.1c19430 (PMC9100494; doi:10.1021/acsami.1c19430)
Supplement: Supplementary file 1 — am1c19430_si_001.pdf [file am1c19430_si_001.pdf]

**Supporting Information for:**

# Dual-grafting of microcrystalline cellulose by tea polyphenols and cationic ε-polylysine to tailor a structured antimicrobial soy-based emulsion for 3D printing

*Mahdiyar Shahbazi*[†*], *Henry Jäger*[†*], *Rammile Ettelaie*[‡]

†Institute of Food Technology, University of Natural Resources and Life Sciences (BOKU), Muthgasse 18, 1190 Vienna, Austria

‡Food Colloids and Bioprocessing Group, School of Food Science and Nutrition, University of Leeds, Leeds, LS2 9JT, UK

# Table of Contents


**S.1. Materials** ........ 3
**S.2. Characterization of grafted MCC conjugates** ........ 3
S.2.1. Determination of Grafting Degree ........ 3
S.2.2. Fourier-transform infrared spectroscopy (FTIR) ........ 4
S.2.3. Solid-state $^{13}C$ NMR spectroscopy ........ 5
S.2.4. X-ray photoelectron spectroscopy (XPS) ........ 5
S.2.5. X-ray diffraction (XRD) ........ 5
S.2.6. Water contact angle ........ 6
S.2.7. Surface charge density ........ 6
S.2.8. Scavenging activity on DPPH free radicals ........ 7
S.2.9. Total antioxidant capacity ........ 7
S.2.10. Antimicrobial properties ........ 8
**S.3. Preparation of SPI-based Pickering emulsion** ........ 8
**S.4. Characterization of soy protein-based ink** ........ 9
S.4.1. Emulsion stability by vertical laser profiling ........ 9
S.4.2. Particle size measurement ........ 10
S.4.3. Confocal laser scanning microscopy (CLSM) ........ 10
S.4.4. Rheological experiment ........ 10
S.4.5. Creep and creep-recovery test ........ 11
S.4.6. Three interval thixotropy test (3ITT) ........ 11
S.4.7. Time evolution of adsorption kinetics and viscoelastic modulus ........ 12
S.4.8. Quartz crystal microbalance with dissipation monitoring (QCM-D) ........ 13
S.4.9. The 3D printing of prepared Pickering emulsion gels ........ 14
S.4.10. Statistical analysis ........ 14
**S.5. Characterization of 3D printed objects** ........ 14
S.5.1. Printing performance measurement ........ 14
S.5.2. Morphology structure ........ 14
S.5.3. Mechanical strength of the 3D printed objects ........ 15
**S.6. RESULTS AND DISCUSSION** ........ 15
S.6.1. FTIR measurement ........ 15
S.6.2. $^{13}C$ NMR spectroscopy ........ 16
S.6.3. XRD pattern ........ 16
S.6.4. Antioxidant activity of “Pickering emulsion gels” and “3D printed objects” ........ 17
S.6.5. Antimicrobial activity ........ 17
S.6.7. Toxicity ........ 18
**S.7. Supporting Information for Figures:** ........ 18
**S.8. Supporting Information for Tables:** ........ 22
**References:** ........ 23

## S.1. Materials

Soy protein isolate (SPI) (moisture: 4.83%, fat: 0.32%, protein: 92.88%, ash: 3.40%, pH: 7.09, and viscosity of 1% wt. solution: 10 cP) was obtained from Archer Daniels Midland Company (ADM, Decatur, IL). Microcrystalline cellulose (MCC) Avicel® PH-101 was purchased by Sigma (Sigma-Aldrich GmbH, Sternheim, Germany). Tea polyphenols (TP) isolated from tea leaves (containing 87.5% EGCG, 5.2% ECG, 3.4% EGC, 2.1% EC, and 0.5% C, respectively) were purchased from the Yuanye Bio-tech. Co. Ltd. (Shanghai, China). The cationic antimicrobial ε-polylysine (ε-PL) was purchased from Nanjing Sai Taisi Biotechnology Co., Ltd. (Nanjing, China). Folin-Ciocalteau's phenol reagent and 2,2-diphenyl-1-picrylhydrazyl (DPPH) were purchased from MP Biomedicals (Irvine, CA). L-ascorbic acid and hydrogen peroxide ($H_2O_2$) were obtained from Sigma (Sigma-Aldrich GmbH, Sternheim, Germany). The HEPES (4-(2-hydroxyethyl)-1-piperazineethanesulfonic acid) was purchased from Sigma-Aldrich (Steinheim, Germany). Free radical 2,2-diphenyl-1-picrylhydrazyl (DPPH) and 2,4,6-Tripyridyl-S-triazine (TPTZ) were supplied from Sigma-Aldrich (St. Louis, MO). All other reagents used were analytical grade without further purification. Dimethyl sulfoxide (DMSO) and tetrabutylammonium fluoride (TBAF) were purchased from Sigma-Aldrich (Steinheim, Germany).

## S.2. Preparation and characterization of grafted MCC conjugates

### S.2.1. Dual-grafting modification of MCC

MCC was completely dispersed in distilled water (100 g $L^{-1}$) and stirred using a magnetic stirrer for 120 min at ambient temperature. Then, the MCC suspension was sheared using a high-speed rotor-stator device (Ultra-Turrax, IKA* T25 digital, Germany) for 20 min, which generated shear force at a shear rate of 210 $s^{-1}$ (5690 G-force). After completing the process, the sheared MCC was collected and dried in an oven at 40 °C for 36 h. After that, the dried MCC was ground to disrupt the clumps, and filtered by a sieve to attain a particle size of 20 μm.

Initially, a free-radical grafting method was applied to graft the tea polyphenols (TP) onto the MCC backbone. In this case, the pre-treated MCC (5 g) was dispersed in 100 mL of Milli-Q water (5 wt.%) and stirred through

a high-speed rotor-stator device (Ultra-Turrax, IKA* T25 digital, Germany) at 50°C for 120 min to obtain a homogeneous dispersion. Next, 5 mL $H_2O_2$ (1.0 M) containing 0.08 g of ascorbic acid was introduced to the MCC-based dispersion. Then, the MCC dispersion containing redox initiator compounds was homogenized via an ultrasonic cleaning device (Bandelin 400, Berlin, Germany), operating at 15 kHz for 2 min. Based on the preliminary grafting measurement (Shahbazi, Jäger, & Ettelaie, 2022), an appropriate ratio of TP (1.5 wt.%) to MCC (1 wt.%) was poured into the reaction vessel and vigorously stirred at 25 °C for 48 h to ensure complete hydration. The product was labeled as MCC-*g*-TP.

In the final step, the stock solutions of ε-PL (1.0 w/v %, pH 5) and TP coated-MCC conjugate (2.0 w/v %, pH 5) were prepared by dispersing suitable quantities of powdered ingredients into double Milli-Q water, subsequently pH adjustment via the introduction of NaOH or HCl solution. The reaction was accomplished for 18 h in ambient temperature and atmosphere. Afterward, the product (MCC-*g*-TP-*g*-PL) was centrifuged (Eppendorf centrifuge 5417R, Hamburg, Germany) at 1409 G-force for 30 min in the ambient condition and washed four times with Milli-Q water. Finally, the supernatant was dried using a freeze-dryer device (Christ Alpha 1-2LD plus, Germany) to produce a well-separated particle. Blank MCC, as a control, was obtained in similar circumstances albeit without the polyphenols. Likewise, an MCC/TP conjugate was produced without a redox initiator compound. In this case, a ratio of 1.5:1 (TP to MCC) was mixed and stirred at 25 °C for 48 h to monitor the efficiency of the free-radical reaction.

### S.2.2. Fourier-transform infrared spectroscopy (FTIR)

The transmission infrared spectra of the pristine MCC, MCC/GA, MCC-*g*-TP, and MCC-*g*-TP-*g*-PL were identified with an FTIR spectrometer (Jasco FT/IR6200, Tokyo, Japan) to ensure the grafting process The solid samples needed for the FTIR assay were obtained in the pellet form by blending 10 mg of each sample with 100 mg of dry KBr. Next, the samples were transferred to pellets to scan the spectral area at the wavenumber ranges of 400 and 4000 $cm^{-1}$, in which 50 scans were recorded with 1 $cm^{-1}$ resolution.

S.2.3. Solid-state $^{13}C$ NMR spectroscopy

To further verify the surface grafting of MCC structure upon introducing TP and ε-PL, solid-state $^{13}C$ NMR was performed through a Bruker spectrometer (AvanceIII 500, Bruker, Ettlingen, Germany) equipped with a 4-mm MAS (magic angle spinning) probe, where frequency for carbons and protons was 75.46 and 300.13 MHz, respectively. The external reference was glycine was utilized aimed at the $^{13}C$ spectra and to set the Hartmann-Hahn matching condition in the cross-polarization experiments. The spectrum of each sample was obtained with the ramp {1H} → {13C} cross-polarization (CP)/MAS pulse sequence using the proton decoupling upon acquisition. The recycling period was 10 s and a contact time of 3 ms during CP was adjusted for all experiments. The SPINAL64 (small phase incremental alternation with 64 steps) sequence was employed for heteronuclear decoupling upon acquisition with a proton field H1H satisfying ω1H/2π = γHH1H = 62 kHz. The spinning rate for all samples was 10 kHz.

S.2.4. X-ray photoelectron spectroscopy (XPS)

The XPS assay was also conducted to ensure the surface grafting modification of MCC. The experiments were conducted through a Kratos Axis spectrometer (Ultra Kratos Analytical, Manchester, UK) via a monochromatic Al Kα source and a 180° hemispherical electron energy analyzer, working at pass energy of 65 eV. Sample preparation for XPS measurements was performed under an inert (argon) atmosphere through a fast entry lock attached to the preparation chamber. The MCC samples were dissolved in DMSO/TBAF at 60 °C and spin-coated at 3000 rpm onto cleaned silicon wafers separately. The silicon wafers were washed with toluene (twice) and acetone, then dried in an oven at 100 °C before spin-coating any samples. The step size was 0.1 eV with a dwell time of 1000 ms. The analyzed zone was adjusted at a region of (300 × 700) $\mu m^2$. A Shirley baseline was employed aimed at the subtraction of the background, and Gaussian/Lorentzian (70/30) peaks were applied for spectral decomposition. Spectra were analyzed through Vision software supplied from Kratos (Vision 2.2.2, Ultra Kratos Analytical, Manchester, UK).

S.2.5. X-ray diffraction (XRD)

The XRD diffractogram was obtained through an X-ray diffractometer (Shimadzu XRD 7000, Tokyo, Japan) with Cu Kα irradiation. The samples were exposed to the X-ray beam at 2θ angles ranging from 2° to 60°

running at 45 kV and 40 mA, employing Cu Kα radiation (λ = 1.541 Å) at a speed of 2° $min^{-1}$. The tested MCCs were initially conditioned in a desiccator including a saturated sodium chloride solution (relative humidity of 75.1%) at ambient temperature for two days to reach a moisture content of 13 g/100 g. To evaluate the relative crystallinity degree (*RCD*), total curve area ($I_t$) and the area under the peaks ($I_p$) were determined using the software offered by Shimadzu, and *RCD* was measured from Eq. S-1:

$$\mathrm{RCD}\ (\%) = \left(\mathrm{I}_p / I_t\right) \times 100 \quad \text{(S-1)}$$

S.2.6. Water contact angle

The contact angle (CA) was obtained by an OCA 20 contact angle meter (Dataphysics Instruments GmbH, Filderstadt, Germany) using the sessile drop approach in natural light. A uniform thin film was fabricated using KW-4A spin-coater (CHEMAT Technology Northridge, CA) by spin coating of 2.0 wt % pristine and modified MCCs (in toluene) suspensions onto the silicon wafers at a shear rate of 210 $s^{-1}$ (5690 G-force) for 1 min, followed by heat treatment at 90 °C for overnight. The obtained films were then sectioned (4 × 6) $cm^2$ and placed on a horizontal movable stage. A drop (5 μL) of Milli-Q water with a syringe (10 μL, Hamilton, Switzerland) was deposited centrally on the surface of the films. The data were analyzed by the software offered by Dataphysics Instruments.

S.2.7. Surface charge density

The surface charge density of pristine MCC and modified MCCs was determined by conductivity titration. The method was in line with Araki's approach with some modifications (Araki, Wada, & Kuga, 2001). Each suspension (20 mL, 0.1 wt%) was mixed with 30 mL of 1 mM NaCl, followed by adjusting the pH to 3.0. The mixtures were then stirred for 10 min and titrated with 0.02 M NaOH under stirring. A curve was made to reflect the relationship between solution conductivity and the volume of NaOH. The charge density of the carboxylate groups was calculated according to the following equation:

$$\text{Charge density (mmol/g dry cellulose)} = V \times c / m \quad \text{(S-2)}$$

where $V$ is the NaOH volume consumed by weak acid (mL), $c$ is the NaOH concentration (mol $L^{-1}$), and $m$ is the dry weight of MCC.

S.2.8. Scavenging activity on DPPH free radicals

The stock DPPH solution was obtained by introducing 5.0 mg of DPPH in methanol (100 mL). The aqueous suspensions/solutions of pristine MCC, TP, ε-PL and grafted MCC conjugates were individually prepared by dispersing 50.0 mg of each sample in 100 mL of Milli-Q water and stirred for 60 min. Then, the DPPH solution ($2\times10^{-4}$ M, 100 μL) was blended with these aqueous suspensions/solutions (100 μL). The resulting mixtures were shaken vigorously and were incubated at ambient temperature in the dark for 1 h. Next, the reactants were centrifuged (Eppendorf centrifuge 5417R, Hamburg, Germany) at 4000 G-force for 5 min. Then, the absorbance was measured at 517 nm using a spectrophotometer (UVIDEC-50, JASCO Corporation, Tokyo, Japan). The scavenging effect of DPPH radical was measured as follows:

$$\text{Scavenging effect (\%)} = [1 - \frac{(A_{s\_517nm} - A_{b\_517nm})}{A_{0\_517nm}}] \times 100 \quad \text{(S-3)}$$

where $A_{0\_517nm}$ is the absorbance of the control (using deionized water instead of the sample), $A_{s\_517nm}$ is the absorbance of the samples mixed with reaction solution, and $A_{b\_517nm}$ is the absorbance of the sample under the same condition as $A_{s\_517nm}$, but ethanol was used instead of ethanol solution of DPPH.

S.2.9. Total antioxidant capacity

The ferric-reducing antioxidant potential was used to determine the total antioxidant capacity. The ferric reducing antioxidant potential working solution was provided by mixing 100.0 mL of sodium acetic buffer (0.3 M, pH 3.6), 10.0 mL of TPTZ (10 mM, dissolved in 40 mM HCl), and 10.0 mL of $FeCl_3$ solution (20 mM) together. The mixture containing 100 μL of sample and 200 μL of the ferric reducing antioxidant potential solution was incubated at room temperature for 20 min, and the Abs was detected at 593 nm.

$$\text{Total Antioxidant Capacity (\%)} = [1 - \frac{(A_{s\_593nm} - A_{b\_593nm})}{A_{0\_593nm}}] \times 100 \quad \text{(S-4)}$$

where $A_{0\_593nm}$ is the absorbance of the control (using deionized water instead of the sample), $A_{s\_593nm}$ is the absorbance of the samples mixed with a working solution, and $A_{b\_593nm}$ is the absorbance of the sample under the same condition as $A_{s\_593nm}$, but ethanol was used instead of working solution.

### S.2.10. Antimicrobial properties

Three bacteria cocktails containing identical populations of 5 trial strains/serovars were utilized in the antimicrobial assessment, including (1): S. enterica cocktail: S. Montevideo, S. Gaminara, S. Agona, S. Michigan, and S. Saint Paul; (2): E. coli O157:H7 cocktail: H1730, K3995, F4546, 658, and 932; (3): L. monocytogenes cocktail: LM1, LM2, 310, Scott A, and V7. Tryptic soy broth (TSB) was employed for S. enterica and E. coli O157:H7, and Tryptic soy broth supplemented with yeast extract (TSBYE) was applied for the growth of L. monocytogenes.

A disk diffusion technique was applied to assess the antimicrobial features of samples. First, the MCC film variants were fabricated by the spin coating method (see Section 2.3.5). Then, the tryptic soy agar (TSA) or TSA supplemented with yeast extract (TSAYE, for L. monocytogenes) plates were spread with a 100 mL culture with $10^5$ CFU $mL^{-1}$ of bacteria cocktail. Two circular discs of each film specimen (10 mm) were transformed into each plate. After incubation for 24 and 48 h at 32 °C (L. monocytogenes) or 37 °C (E. coli O157:H7 and S. enterica), the diameter (mm) of inhibition zones was determined through a ruler. The mean values of inhibition zone diameters from two films with two discs each (n = 8) were reported.

## S.3. Preparation of SPI-based Pickering emulsion

The SPI aqueous dispersion was made by dispersing SPI powder (25.0 g) into part of the citrate phosphate buffer (pH 5.6, 60 mL), with the rest of the water being used for the grafted MCC conjugates. Next, the SPI-based dispersion was gently stirred at 40 °C for 60 min through a magnetic heater stirrer. At the same time, 10% (v/v) sunflower oil was incorporated into the SPI-based dispersion with a burette. The obtained emulsions were stirred by an Ultra-Turrax at a speed of 210 $s^{-1}$ (5690 G-force) for 5 min. Separately, a stock suspension of the grafted MCC conjugates was made by dispersing weighed amount (70 wt.%) of the powdered grafted MCCs into the same buffer (pH 5.6, 40 mL) and mixed with a high-speed rotor-stator device (Ultra-Turrax T25D IKA, Germany) at a shear rate of 400 $s^{-1}$ (20664 G-force) for 10 min at ambient temperature. Then, the grafted MCC suspension was gently stirred overnight at room temperature. The pH of this suspension was then adjusted back to pH 5.6.

An O/W emulsion was prepared by blending 10 wt.% sunflower oil and 90 wt.% aqueous SPI-based dispersions (25.0 wt.% SPI, pH 5.6) using a high-speed blender (Ultra-Turrax T25D IKA, Germany) for 5 min. This coarse emulsion was homogenized by a two-stage high-pressure Microfluidizer processor (M110-PS, Microfluidics international Corp., Newton, MA) with 1800 psi at the first stage and 700 psi at the second stage. The full-fat stabilized emulsion regarded as control hereafter (10 wt.% sunflower oil, 25.0 wt.% SPI, pH 5.6) was employed to develop reduced-fat emulsions. After that, a 60% reduced-fat SPI-based Pickering emulsion gel was prepared by replacing oil with the stock suspensions of the pristine MCC (SP/MCC) or MCC-*g*-TP (SP/MCC-*g*-1), and MCC-*g*-TP-*g*-PL (SP/MCC-*g*-2) (70 wt.%, pH 5.6). The reduced-fat emulsions contained 4 wt.% sunflower oil and 4.2 wt.% of pristine MCC or grafted micro-conjugate variants.

## S.4. Characterization of soy protein-based ink

### S.4.1. Emulsion stability by vertical laser profiling

The global Turbiscan stability index (*TSI*) parameter is commonly employed to determine emulsion stability, accounting for diverse storage processes of emulsion (particle coalescence and settling processes). The stability experiment was performed by vertical laser profiling using a Turbiscan Lab Expert stability analyzer (Formulaction, Toulouse, France) for 180 min under ambient conditions. The emulsions stability was carried out according to multiple light backscattering of a pulsed near-infrared light (880 nm). First, the inks were moved to a tested bottle attaining a height of 42 mm and scanned the entire height of the inks every 4 h for 7 times, and the differences in the back-scattering and transmission light were detected. The transmittance detector received the light that passed through the dispersion at an angle of 180° concerning the source, while the backscattering (BS) detector received the light scattered backward by the emulsion at an angle of 45°. The emulsion stability was determined with the *TSI* as follows:

$$TSI = \sum_{i=1}^{n}(x_i - x_{BS})^2 \Big/ n-1 \qquad \text{(S-5)}$$

where, $\lambda^*$ is the photon transport mean free path, $\varphi$ is the particles' volume fraction, $d$ is the particles' mean diameter, and $g$ and $Qs$ are the optical parameters assumed with the Mie theory. The $\chi_i$ is the average backscattering for each minute during the experiment, $\chi_{BS}$ is the average $\chi_i$, and $n$ is the number of scans.

S.4.2. Particle size measurement

The inks were diluted to a droplet level of about 0.005 wt.% with a citrate phosphate buffer (pH 5.6) to avoid multiple scattering impacts. Each dispersion was stirred gently at room temperature to ensure the inks were homogenous. The droplet sizes and particle size distribution of the inks were measured with a laser diffraction device (MS2000, Malvern Instruments Ltd., Worcestershire, UK), measuring the size based on the scattering of a monochromatic beam of laser light (k = 632.8 nm). The droplet size was specified as the volume mean diameter $d_{(4,3)} = (\sum n_i d_i^4 / \sum n i d_i^3)$, where $n$ is the number of droplets with diameter $d_i$.

S.4.3. Confocal laser scanning microscopy (CLSM)

The interfacial framework and network structure in the continuous phase of the soy protein-based inks were imaged via an FV-300 confocal laser scanning microscopy system (CLSM, Olympus, Tokyo, Japan) coupled with an Olympus IX71 inverted microscope and an argon-ion laser. The inks (5 mL) were stained with the defined level of Nile Blue A (1.0%, w/v) in deionized water, or the blend of Nile Blue A (1%, w/v) and Nile Red (0.1%, w/v) in 1,2-propanediol (including deionized water, 20 μL $g^{-1}$) to mark the protein and/or modified MCC and oil droplet, respectively. The level of both Nile blue A and Nile red solution was 0.01% (w/v). The excitation wavelengths of fluorescent in the system were 488 nm (Nile red) and 633 nm (Nile blue A). The ink microstructures were imaged at ambient temperature directly after staining. All images were obtained at 40× magnification and processed using Olympus Fluoview software (version 2.1, Olympus, Tokyo, Japan).

S.4.4. Rheological experiment

The rheological behavior of ink samples was characterized by AR 2000ex rheometer (TA Instruments, New Castle, DE) using a parallel plate geometry (diameter 40 mm, gap 1 mm). The oscillatory strain sweep (0.1–100%, 1 Hz) was performed to attain the limitation of the linear viscoelastic region (*LVR*). Besides, the frequency sweep test (0.1–100 Hz) was accomplished in the LVR ($\gamma$ = 1%). All measurements were performed at 25 °C.

To evaluate the steady rheological properties, the shear stress ($\tau$) was measured as a function of increasing shear rate ($\gamma$ ) from 0.1–100 $s^{-1}$. The best equation was selected via statistical analysis and the rheological

variables were measured with the optimum model. Hence, the consistency index, flow behavior index, and yield stress values were obtained by fitting the Herschel-Bulkley model to the data:

$$\tau = \tau_0 + K\dot{\gamma}^n \quad \text{(S-6)}$$

where $\tau$ is shear stress (Pa); $\tau_0$ is the yield stress (Pa); *K* is the consistency index (Pa $s^n$); $\gamma$ is the shear rate ($s^{-1}$), and *n* is the flow behavior index.

S.4.5. Creep and creep-recovery test

The creep and creep-recovery measurements were performed to evaluate the compliance level in the creep and recovery stages via AR 2000ex rheometer (TA Instruments, New Castle, DE). First, a stress sweep (1 Hz, 0.1-1 Pa) was accomplished (Supporting Information, Figure S-6) to evaluate the oscillatory yield stress (*G′ (τ) = G″ (τ)*), and then the obtained shear stress values were considered as about 50% of the yield stress. The inks were moved to a parallel-plate geometry with a diameter of 40 mm and a 1 mm gap at 25 °C. The creep measurement included the application of prompt and constant shear stress within the LVR area, from 0 to 500 s, whereas evaluating the sample deformation between time intervals. Regarding the recovery phase, the applied stress was rapidly removed ($\tau_{applied}$ = 0.0 Pa) and the recovery values were recorded for a further time of 500 s at the same temperature in the creep phase. The calculated strain and recovery were considered as creep compliance and creep-recovery compliance *(J)* (Eq. S-7). The creep-recovery percentages of inks were then obtained according to Eq. (S-8):

$$J(t) = \gamma(t) / \tau_0 \quad \text{(S-7)}$$

$$\text{Percentage recovery} = (J_m - J_e) / J_m \times 100\% \quad \text{(S-8)}$$

where *J(t)* ($Pa^{-1}$) is creep compliance, $\gamma$ is the measured strain, *t* is time, $\tau_0$ is the constant applied shear stress, $J_m$ ($Pa^{-1}$) is the maximum creep, and $J_e$ ($Pa^{-1}$) is the equilibrium creep compliance after recovery.

S.4.6. Three interval thixotropy test (3ITT)

The 3ITT measurement was used to evaluate if the Pickering emulsions are prone to rapid recovery upon being sheared at the large deformations. Perfectly, a material can be shown as an ideal thixotropic structure if the peak viscosity in the third interval recovers at least 70% of its value measured after the initial interval.

The 3ITT contained a three-step shear rate test, where the first one comprised a steady shear rate to recognize the ink reference stage without interrupting the microstructure with a fixed shear rate of 1 $s^{-1}$ for 400 s. This was followed by the second interval, in which a steady shear rate of 80 $s^{-1}$ for 200 s was used to terminate the microstructure of the ink. The third interval included a similar assessment condition as the first interval, gaining the reversible restructuration (speed and degree of recovery) of Pickering emulsions (10).

S.4.7. Time evolution of adsorption kinetics and viscoelastic modulus

An automated drop tensiometer (Tracker-H, TECLIS, Lyon, France) was used to determine the interfacial surface pressure ($\pi$) and adsorption properties of SPI alone or SPI containing MCCs at the O/W interface. A cuvette and syringe were employed as a container for the aqueous phase and oil phase, respectively. Control was Milli-Q water. The O/W interfacial pressure ($\pi$, mM/m) was measured at the ambient condition (continued for 7200 s) using the following equation:

$$\pi = \gamma_0 - \gamma \tag{S-9}$$

where $\gamma_0$ and $\gamma$ (mN/m) are the interfacial tension of water and that of SPI alone or SPI containing MCCs. Besides, the Ward-Tordai equation, valid at the early stages of the diffusion-adsorption process, was used to monitor the changes of $\pi$ versus adsorption time ($t$) (Ward & Tordai, 1946):

$$\pi = 2C_0 k_B T \left( {Dt}/{3.14} \right)^{0.5} \tag{S-10}$$

Here $C_0$ is the concentration of the continuous phase, $k_B$ is the Boltzmann constant, $T$ is the absolute temperature and $Dt$ is the diffusion coefficient of the emulsifier at $t$. The diffusion rate constant ($K_{diff}$) is calculated as the slope of $\pi$ versus $t^{0.5}$.

The drop tensiometer was also used to measure the dynamic interfacial viscoelasticity of SPI alone or SPI containing MCCs at the O/W interface at 25 °C, for a period of 7200 s. The sinusoidal interfacial compression and expansion were performed by changing the drop volume at 10% of deformation amplitude ($\Delta A/A$ = 0.1) within the linear regime with the process involving 5 active cycles and 5 blank cycles. The oscillation

frequency was 0.1 Hz and the volume of droplets was 10 µL. The interfacial modulus (*E*) of the dilatational elasticity was calculated using the following equation:

$$\sigma = \sigma_0 \exp\left(i(\omega t + \delta)\right) \quad \text{(S-11)}$$

$$\Delta A = (\Delta A_0) \exp(i\omega t) \quad \text{(S-12)}$$

$$E = \frac{d\gamma}{d\left(\frac{\Delta A}{A0}\right)} = \frac{d\gamma}{d(Ln(A))} \quad \text{(S-13)}$$

where $\sigma$ is the dilatational stresses at times *t*, $\delta$ is the phase angle between the stress and strain oscillations, $\Delta A$ is the change in the interfacial areas of the drop at times t while $\Delta A_0$ represents the amplitude of the imposed change, $A_0$ is the initial surface of the droplet and finally, $\pi$ is the interfacial pressure. The complex surface dilatational modulus (*E*, where $E=E_d+ iE_v$) includes the elastic ($E_d$) and the viscous ($E_v$) moduli.

S.4.8. Quartz crystal microbalance with dissipation monitoring (QCM-D)

A QCM-D (Q-Sense E4 AB, Gothenburg, Sweden) was used to evaluate the adsorption properties of SPI-based ink variants with the ability to detect variations of resonance frequency ($\Delta f$) and energy dissipation ($\Delta D$) of QCM-D sensors. The gold sensors with a fundamental resonance frequency of 4.95 MHz (QSX 301) were cleaned and coated with a monolayer of sunflower oil by spin coating at 2000 rpm for 2 min with the oil solution (0.1 wt% in chloroform). The experiment was conducted at a flow rate of 50 µL $min^{-1}$ at ambient conditions, and the Milli-Q water was used to establish a signal baseline. The Sauerbrey equation was used to make a relationship between $\Delta f$ and the coupled mass ($\Delta m$):

$$\Delta m = -C\left({}^{\Delta_f}/_{n}\right) \quad \text{(S-14)}$$

In the above equation, *C* is a mass constant depending on the physical property of the sensor (17.7 ng $cm^{-2}$ $Hz^{-1}$ for a 5 MHz crystal), and *n* is the resonance number of the sensor (n = 1, 3, 5, 9, 11, 13). For the sake of simplicity, only one harmonic (n = 5) was presented in the results here. In the current work, the fifth

harmonic was employed to measure the dissipation shifts and frequency, as well as to obtain the corresponding mass change.

### S.4.9. The 3D printing of prepared Pickering emulsion gels

The prepared soy protein-based inks were printed through an extrusion-based 3D printer (nScrypt-3D-450, nScrypt, Orlando, FL), connected to a syringe pump (PHD Ultra; Harvard Apparatus Holliston, MA). A special cube shape was modeled by application of computer-aided design software (AutoCAD; Autodesk Inc., San Rafael, CA), and converted to an STL file. Each Pickering emulsion gel was then printed in a size of (5×5×5) $cm^3$ as a cube through a needle diameter of 1 mm with an extrusion flow speed of 50 mL $min^{-1}$ at an ambient temperature on a special plastic surface.

The print paths were provided through the creation of the G-code files to control the XYZ direction instruction of the printer, developed by the open-source CAM software Slic3r (slic3r.org, consulted on December 2020) from the STL file. The printable soy protein-based inks were poured into a stainless-steel cartridge (10 mL) and stirred with a Vortex mixer (Fisher Scientific, Ontario, Canada) for 10 min to remove the air bubbles from the ink. The layer height was set at 1 mm, proposing that the nozzle tip was elevated by that value upon completion of the fabrication of each layer, continuing until the suitable 3D architectures were printed.[10] The height of the tip was increased by 1.1 mm after the deposition of each layer. The number of deposited layers was 8 and the width of the tip was 1 mm (Supporting Information, Table S-1).

### S.4.10. Statistical analysis

All instrumental experiments were carried out as triplicate determinations and the mean and standard deviation of the data were reported. Analysis of variance (ANOVA) was utilized the determination of the main effects of the examined independent factors and their interactions with the instrumental data. Duncan's multiple range test was applied to separate means of data when significant differences ($P < 0.05$) were observed.

## S.5. Characterization of 3D printed objects

### S.5.1. Printing performance measurement

Each 3D printed object was transferred into a specific chamber (20 × 20 × 20) $cm^3$ for taking photos using a digital camera (Alpha 7M3 E-Mount, Full-Frame Mirrorless, 24.2 MP, Sony, Tokyo, Japan). The printing performance of 3D printed architectures was accomplished by determining through a digital caliper (Mitutoyo, Absolute Digimatic, Tokyo, Japan).

### S.5.2. Morphology structure

The influences of surface-active MCCs on the morphological structure of the 3D printed objects were captured through a variable-pressure scanning electron microscope (VP-SEM Quanta 200 FEG SEM, FEI Company, Eindhoven, Netherlands) to produce high-resolution images with a high-depth of field. Initially, each 3D construct was cut into a precise size of (15 × 15 × 15) $mm^3$. Next, the sectioned 3D printed samples were mounted on a Peltier-cooled stage with a temperature of −10 °C to avoid thermal damage. The nitrous oxide was utilized as an imaging gas that offered a pressure of 50.7 Pa. The microstructures of each 3D construct were obtained through a solid-state backscatter detector via an accelerating voltage of 20 kV.

### S.5.3. Mechanical strength of the 3D printed objects

Mechanical assay for tensile strength of dumbbell-shaped- 3D structures (10 mm gauge length, 2 mm width, 2 mm thickness) was performed at 100 mm $min^{-1}$ through Instron 3366 electronic universal testing machine (Instron Corporation, MA) The elastic modulus ($E$) of 3D printed samples was determined by the average slope over 10 – 30 % of strain from the stress-strain curve. The fracture energy ($\Gamma$) and toughening mechanism of the 3D printed objects were evaluated as follows. (1) Each loading-unloading cycle was applied to the 3D printed constructs under a tensile strain lower than their corresponding yielding strains. (2) The successive and progressive stretches, where each specimen was stretched to different strains in the first loading and then relaxed to zero force, followed by the second loading. The $E_{2nd}/E_{1nd}$ and $\Gamma_{2nd}/\Gamma_{1nd}$ were determined and used to evaluate the effect of various stretches on the fracture process and toughening mechanism for the 3D structures. For the recovery experiments, the notched samples were tested by a cycle of loading-unloading at a fixed strain ($\varepsilon$ = 400%). Then, the deformed and relaxed notched samples were

sealed in a polyethylene bag and stored in a water bath of 90 °C. Finally, the specimens were taken out at different time intervals and cooled down to room temperature for tensile tests again.

## S.6. RESULTS AND DISCUSSION

### S.6.1. FTIR measurement

The FTIR spectra of pristine MCC, MCC/TP, MCC-*g*-TP, and MCC-*g*-TP-*g*-PL are depicted in Figure 1a. The typically pronounced vibration of pristine MCC presented typical bands of cellulose I centered around 2500-3750 $cm^{-1}$ and 700-1800 $cm^{-1}$. The peak at about 3380 $cm^{-1}$ was also related to the stretching of the -OH group. The other representative bands were comprised of C-H stretching (2953 $cm^{-1}$); asymmetric stretching vibration of -COOH groups (1637 $cm^{-1}$); $-CH_2$ symmetrical bending (1426 $cm^{-1}$); cellulose C-O-C bridges (1075 $cm^{-1}$); ether C-O-C functionalities (962 $cm^{-1}$); and the band at 876 $cm^{-1}$, which is typical of β-linked glucose polymers. The FTIR spectrum of the MCC/TP (with no redox initiator compound) was found similar to the pristine MCC, with no shift in characteristic peaks and emergence of a new peak.

Compared to pristine MCC, the -OH stretching (~ 3350 $cm^{-1}$) was reduced in MCC-*g*-TP, representing that conjugation reactions happened at the hydroxyl sites on the MCC backbone. Besides, the C-H stretch vibration of $-CH_3$ (~ 2950 $cm^{-1}$) was disappeared, signifying a possible hydrogen interaction occurred between the hydrogen of $-CH_3$ or -OH of the MCC backbone and oxygen from -OH groups of TP. A new strong carbonyl stretching vibration at about 1860 $cm^{-1}$ (C=O) was easily identified. Another difference between the pristine MCC and MCC-*g*-TP was the appearance of the band corresponding to the C-O stretching at about 1340 $cm^{-1}$. Besides, there was an emergence of a band at about 690 $cm^{-1}$ caused by the distortion vibrations of benzene rings. These observations suggested the interactions between MCC and TP through free-radical reaction.

### S.6.2. $^{13}C$ NMR spectroscopy

The dual-grafting treatment of pristine MCC was further qualitatively verified by solid-state $^{13}C$ NMR spectroscopy (Figure 1b). The pristine MCC spectrum exposed the typical peaks of cellulose I, *i.e.,* C1: 111.2 ppm, C4: 97.8 ppm, C4': 94.7 ppm, cluster C2-C3-C5: 71.7-79.2 ppm, C6: 70.2 ppm, and C6': 65.3 ppm (5). Again, the MCC/TP (with no redox initiator) presented almost a comparable $^{13}C$ NMR spectrum compared to

pristine MCC (Figure 1b). This specifies that the incorporation of tea phenols did not affect the $^{13}C$ NMR spectrum in the anomeric and non-anomeric proton regions.

S.6.3. XRD pattern

The diffractogram of pristine MCC, and also MCC/TP, exhibited a crystalline structure with strong reflections at 2θ = 14.2° ($d_{001}$ = 5.5 Å), 2θ = 23.0° ($d_{001}$ = 4.9 Å), and 2θ = 35.5° ($d_{001}$ = 4.1 Å), presenting the dominance of cellulose type I in the MCC (Figure 1d). The relative crystallinity degree (*RCD*) of MCC and MC/TP was also obtained at about 77%, which is rather lower than the crystallinity index of about 84% detected by $^{13}C$ NMR.

S.6.4. Antioxidant activity of "Pickering emulsion gels" and "3D printed objects"

In addition to the grafted MCC compounds, the antimicrobial activity of Pickering emulsion gels (including control SPI, SP/MCC, SP/MCC-*g*-1, and SP/MCC-*g*-2 inks), as well as their resultant 3D printed structures were performed. Compared to the grafted MCC-polyphenol conjugates (**Figure 1g** in the main manuscript), the antimicrobial activity and DPPH scavenging activity of the SP/MCC-*g*-1 and SP/MCC-*g*-2 inks or the relevant 3D printed objects were slightly decreased (Figure S-3). The antioxidant data exposed that the control SPI-based ink and SP/MCC and their relevant 3D printed structures had the lowest DPPH scavenging effect, while SP/MCC-*g*-1 ink or its 3D printed sample offered the highest antioxidant activities. These results were not surprising, as the incorporation of tea polyphenols onto MCC could rationally induce a versatile antioxidant character.

S.6.5. Antimicrobial activity

The antimicrobial activity of control SPI, SP/MCC, SP/MCC-*g*-1, and SP/MCC-*g*-2 inks was and their corresponding 3D printed constructs also measured by the disk diffusion experiment (Figure S-4). The control SPI, SP/MCC, and SP/MCC-*g*-1 inks, as well as their relevant 3D printed counterparts, had no inhibitory effect against any of the evaluated microorganisms, where no bacteria growth was detected under the films' discs. In contrast, a considerable inhibition zone diameter was detected concerning discs of SP/MCC-g-2 ink and its 3D printed sample. This antimicrobial effect relates to the ε-PL component, which was also in

accordance with the inhibitory effect of ε-PL alone conducted in the present study (Figure S-4; and **Figure 1g** in the main manuscript). The antimicrobial activity of ε-PL is directly associated with the existence of the positive charge on its protonated guanidine group, disrupting the cell membranes of bacteria without triggering the cell lysis, however, it might also act on the other intracellular targets leading to the lethality of bacteria.

S.6.6. Surface charge density results

The charge density was measured by conductivity titration. The charge density of carboxylate groups of pristine MCC was measured to be 1.23 mmol/g dry cellulose, which was positively correlated with the NaClO dosage used in the process. Similarly, the charge density of MCC/TP was also high with a value of about 1.21 mmol/g dry cellulose. Compared to pristine MCC and MCC/TP, the MCC-*g*-TP and MCC-*g*-TP-*g*-PL had a lower surface charge density with a value of 0.49 and 0.33 mmol/g dry cellulose, respectively.

S.6.7. Toxicity

In the current study, the conformity of the SPI-based ink containing grafted MCC (SP/MCC-*g*-1 ink) with the actual food regulations on the food products was verified by a cytotoxicity test to study the possibility of utilization in the food sector. In this regard, the release of cytoplasmic lactate dehydrogenase (LDH) from the incubated cells can be considered an indicator for cytotoxicity assay (Shahbazi et al., 2016; Shahbazi et al., 2017). The grafted micro-biosurfactant conjugate did not show any evidence of the cytotoxic effects since they could not increase the cytoplasmic lactate dehydrogenase release from L-929 fibroblast cells in contact with the samples.

The same procedure was performed on SPI-based ink containing dual-grafted MCC (SP/MCC-g-2 ink). Cytotoxicity result of this ink was revealed that the extent of cytotoxicity was less than 5.0% after 3, 18, and 36 h when the ink sample was in direct contact with L-929 cells. There was also no significant difference in the release of the LDH compared with the positive control ($P > 0.05$). This indicates that SP/MCC-g-2 ink was non-toxic to the cells.

## S.7. Supporting Information for Figures:

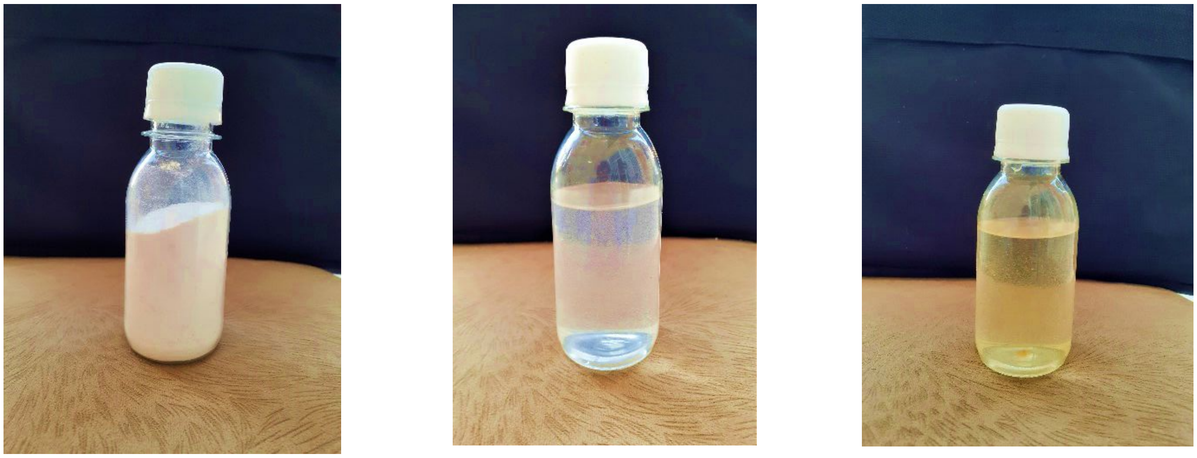

**Figure S-1. Freeze-dried pristine MCC powder (left), redispersed pristine MCC in water (middle), and MCC redispersed in toluene.**

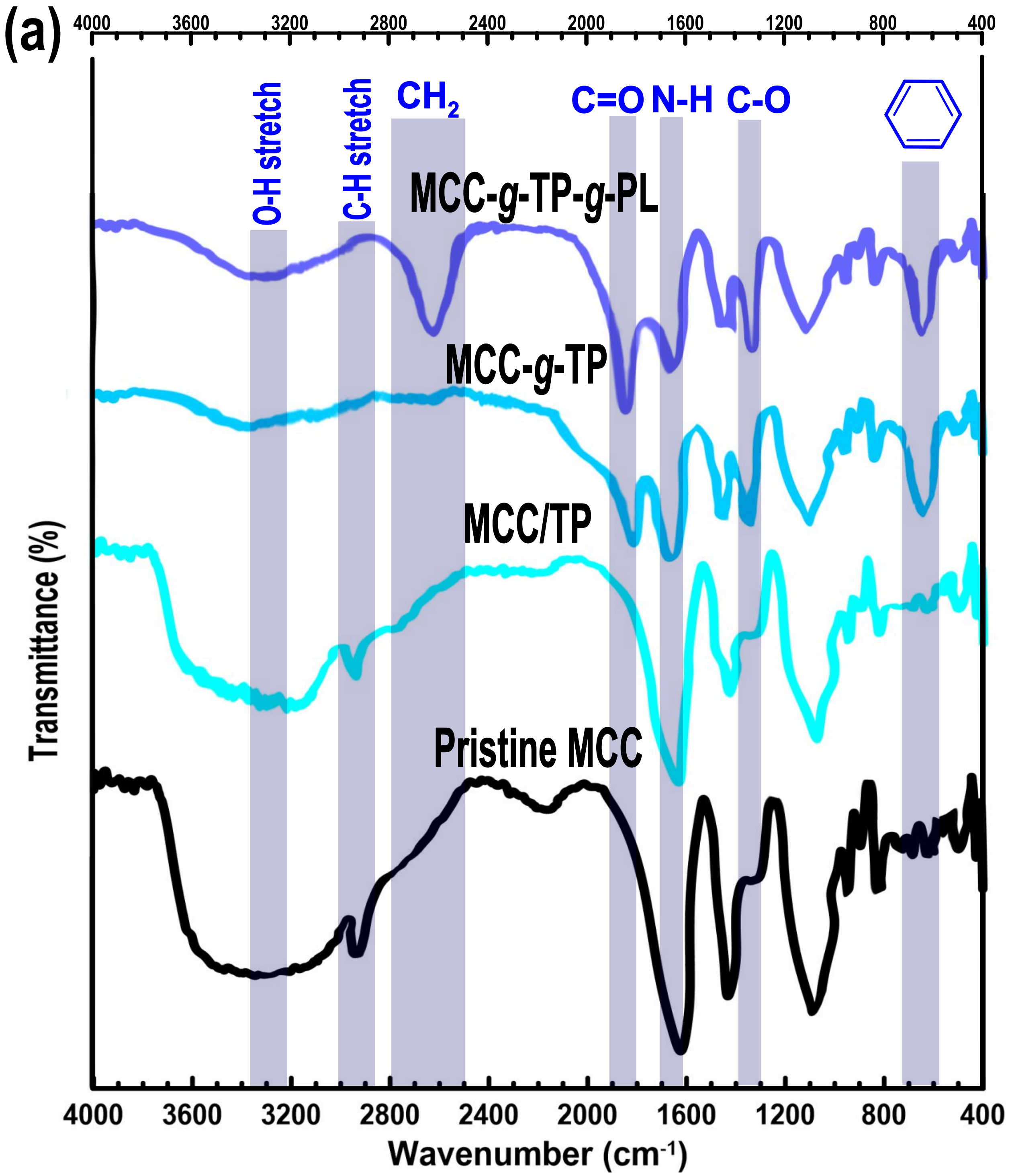


**Figure S-2. FTIR spectra of pristine and modified MCCs.**

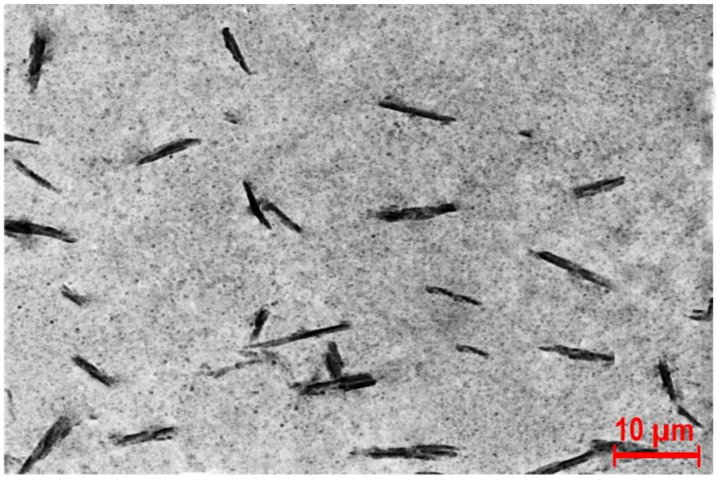


**Figure S-3. SEM of pristine MCC.**

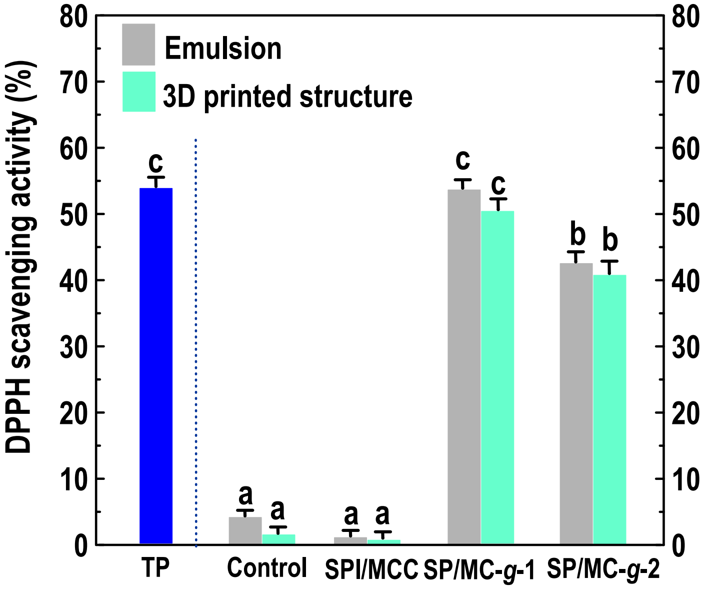


**Figure S-4. DPPH scavenging activity of the Pickering emulsion gel and 3D printed structures of different samples.**

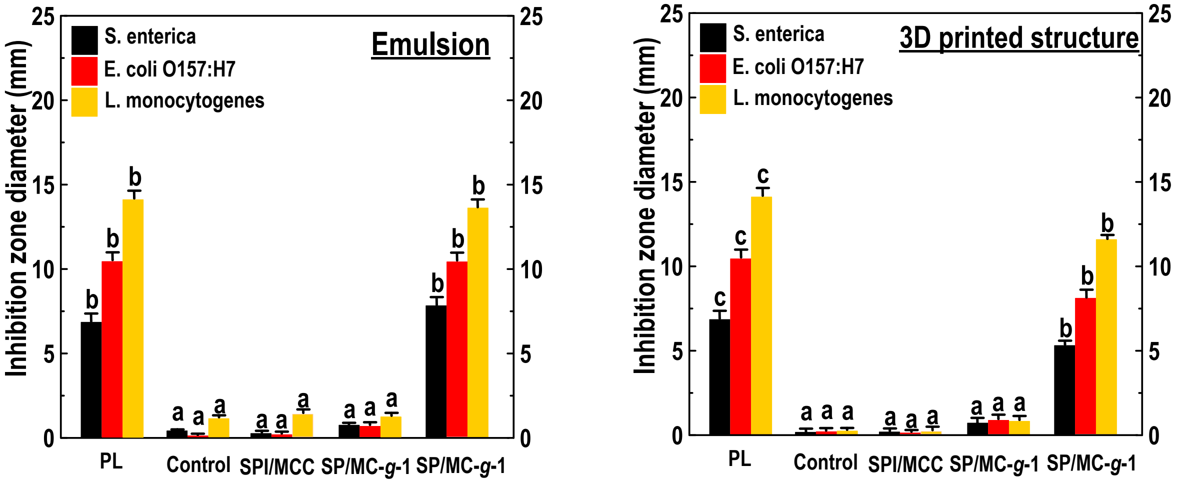


**Figure S-5.** Antimicrobial activity of the Pickering emulsion gel (left) and 3D printed structures (right) of different samples.

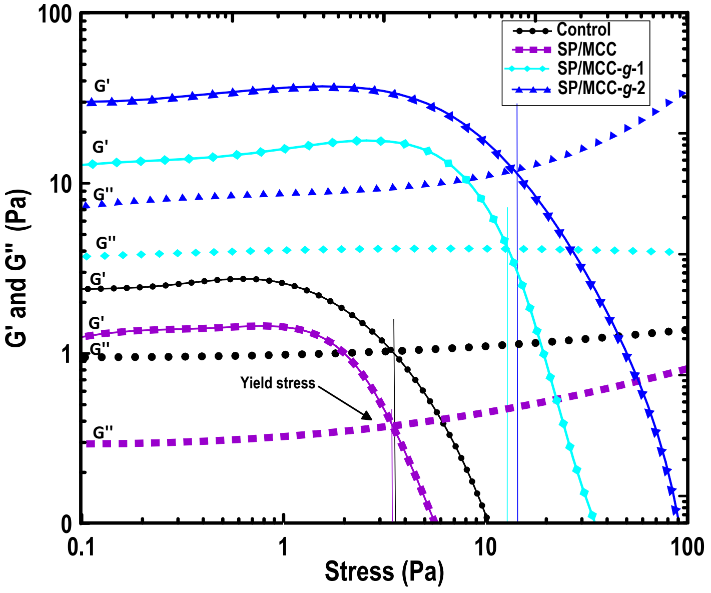


**Figure S-6. Stress sweep test conducted for different ink samples.**

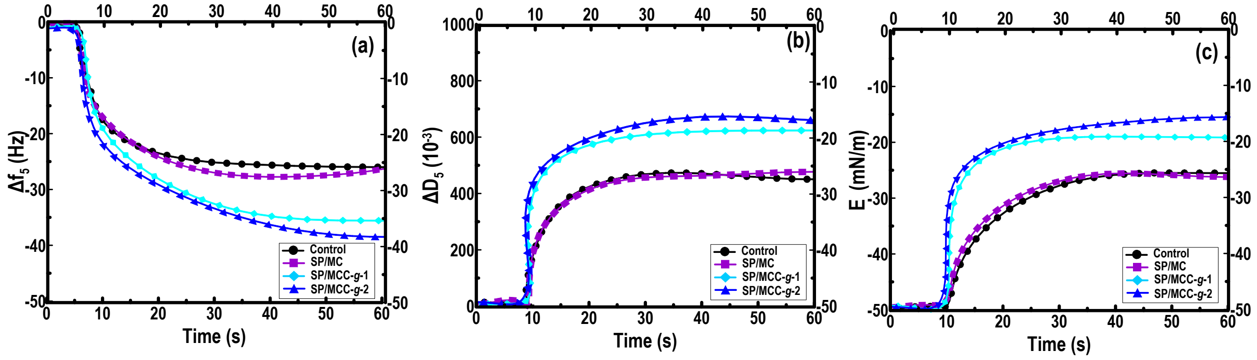

**Figure S-7. The *Δf* (a) and *ΔD* (b) from the fifth harmonic as a function of time for different samples during adsorption and desorption. (c) The coupled mass at the adsorption process measured by the Sauerbrey model through the data from the fifth harmonic.**

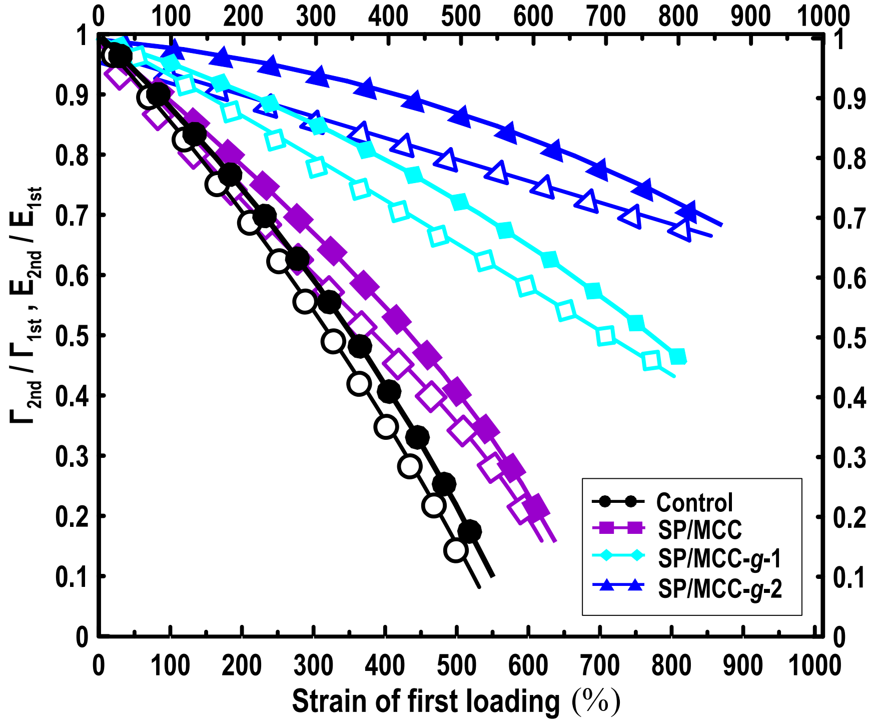


**Figure S-8. The $\Gamma_{2nd}/\Gamma_{1st}$ and $E_{2nd}/E_{1st}$ as a function of the strain of first loading in different printed objects.**

## S.8. Supporting Information for Tables:

**Table S-1**. The printing settings are expressed as Slic3r terms (http://slic3r.com).

| Printing adjusting | Sign | Value | Units | Definition |
|---|---|---|---|---|
| **Nozzle diameter** | D | 1.0 | mm | Nozzle diameter |
| **Layer height** | Z | 1.0 | mm | Layer height |
| **Extrusion flow speed** | Q | 0.50 | mL $min^{-1}$ | Continuous extrusion flow rate provided by the syringe pump |
| **Flow rate** | S | 90 | % | The volume of ink that passes through the extruder |
| **Infill velocity** | V | 15 | mm $s^{-1}$ | Spindle speed during extrusion |
| **Travel velocity** | $V_{travel}$ | 180 | mm $s^{-1}$ | The spindle speed of a jump between the end of one extrusion and the next |

| Perimeter | P | 10 | - | Number of outline layers |
|---|---|---|---|---|
| Infill density | $\rho_{infill}$ | 90 | % | Quantity of material filling the object |

**Table S-2.** Diffusion coefficients ($K_{diff}$) for SPI-based Pickering emulsion variants.

| Sample | $K_{diff}$ |
|---|---|
| Control | 0.947 ± 0.007[c] |
| SP/MCC | 0.912 ± 0.006[b] |
| SP/MCC-*g*-1 | 0.349 ± 0.004[a] |
| SP/MCC-*g*-2 | 0.356 ± 0.002[a] |

[a–c] Means (three replicates) within each column with different letters are significantly different ($P$ < 0.05), Duncan's test.

**Table S-3.** The obtained viscosity, flow behavior index, and yield stress of SPI-based ink variants.

| Sample | Flow behavior index | Consistency index (Pa s$^n$) | Yield stress (Pa) | $R^2$ |
|---|---|---|---|---|
| Control | 0.911±0.057[b] | 7.52±0.07[b] | 1.51±0.19[b] | 0.989 |
| SP/MCC | 0.957±0.052[c] | 4.37±0.08[a] | 1.02±0.44[a] | 0.982 |
| SP/MCC-*g*-1 | 0.509±0.039[a] | 18.44±0.03[c] | 4.22±0.38[c] | 0.973 |
| SP/MCC-*g*-2 | 0.516±0.019[a] | 25.91±0.05[d] | 4.26±0.26[c] | 0.994 |

[a–e] Means (three replicates) within each column with different letters are significantly different ($P$ < 0.05), Duncan's test.

## References:


Araki, J., Wada, M., & Kuga, S. (2001). Steric stabilization of a cellulose microcrystal suspension by poly (ethylene glycol) grafting. *Langmuir*, 17(1), 21-27.

Blainski, A., Lopes, G. C., & De Mello, J. C. P. (2013). Application and analysis of the folin ciocalteu method for the determination of the total phenolic content from Limonium brasiliense L. *Molecules*, *18*(6), 6852-6865.

Shahbazi, M., Ahmadi, S. J., Seif, A., & Rajabzadeh, G. (2016). Carboxymethyl cellulose film modification through surface photo-crosslinking and chemical crosslinking for food packaging applications. *Food Hydrocolloids*, *61*, 378-389.

Shahbazi, M., Jäger, H., & Ettelaie, R. (2022). A Promising Therapeutic Soy-Based Pickering Emulsion Gel Stabilized by a Multifunctional Microcrystalline Cellulose: Application in 3D Food Printing. Journal of agricultural and food chemistry, 70(7), 2374-2388.

Shahbazi, M., Rajabzadeh, G., & Sotoodeh, S. (2017). Functional characteristics, wettability properties and cytotoxic effect of starch film incorporated with multi-walled and hydroxylated multi-walled carbon nanotubes. *International journal of biological macromolecules*, *104*, 597-605.

Ward, A. F. H., & Tordai, L. (1946). Time-dependence of boundary tensions of solutions I. The role of diffusion in time-effects. *The Journal of Chemical Physics*, *14*(7), 453-461.
